# Rainbow: reliable personally identifiable information retrieval across multi-cloud

**DOI:** 10.1186/s42400-023-00146-z

**Published:** 2023-06-03

**Authors:** Zishuai Song, Hui Ma, Shuzhou Sun, Yansen Xin, Rui Zhang

**Affiliations:** 1 State Key Laboratory of Information Security, Institute of Information Engineering, Chinese Academy of Sciences, No. 19 Shucun Road, Haidian District, Beijing, 100084 China; 2 School of Cyber Security, University of Chinese Academy of Sciences, Beijing, China

**Keywords:** Personally identifiable information, Data privacy, Flexible access control, Reliable user revocation, Verification

## Abstract

Personally identifiable information (PII) refers to any information that links to an individual. Sharing PII is extremely useful in public affairs yet hard to implement due to the worries about privacy violations. Building a PII retrieval service over multi-cloud, which is a modern strategy to make services stable where multiple servers are deployed, seems to be a promising solution. However, three major technical challenges remain to be solved. The first is the privacy and access control of PII. In fact, each entry in PII can be shared to different users with different access rights. Hence, flexible and fine-grained access control is needed. Second, a reliable user revocation mechanism is required to ensure that users can be revoked efficiently, even if few cloud servers are compromised or collapse, to avoid data leakage. Third, verifying the correctness of received PII and locating a misbehaved server when wrong data are returned is crucial to guarantee user’s privacy, but challenging to realize. In this paper, we propose Rainbow, a secure and practical PII retrieval scheme to solve the above issues. In particular, we design an important cryptographic tool, called Reliable Outsourced Attribute Based Encryption (ROABE) which provides data privacy, flexible and fine-grained access control, reliable immediate user revocation and verification for multiple servers simultaneously, to support Rainbow. Moreover, we present how to build Rainbow with ROABE and several necessary cloud techniques in real world. To evaluate the performance, we deploy Rainbow on multiple mainstream clouds, namely, AWS, GCP and Microsoft Azure, and experiment in browsers on mobile phones and computers. Both theoretical analysis and experimental results indicate that Rainbow is secure and practical.

## Introduction

Personally identifiable information (PII) (DHS 2021) refers to any information that links to an individual, which is extremely useful for service providers, such as Social Security Numbers, financial records. In particular, securely sharing PII can play an important role in public affairs, e.g., in 2020, the white house attempted to utilize user data (including sensitive PII) of Google and Facebook to fight COVID-19 (https://www.cnbc.com), which brought forth big worries about privacy violations by a single-point failure and was not ever realized.

Building a PII retrieval service over multi-cloud whose access control power is shared among multiple servers seems to overcome the single-point failure issue and enhance the security protection of PII. In particular, a user encrypts and then uploads his PII data to the retrieval service. In a later application, a service provider that is authorized by the PII owner can access the data. Furthermore, the PII owner can decide which subset of his PII can be accessed and forms a specific PII form (PIIF). However, the following three major technical issues need to be addressed.

*Data privacy and flexible fine-grained access control.* A PIIF contains a series of user’s sensitive information, e.g., address, Social Security Number (SSN) and allergens. According to the data privacy laws, such as General Data Protection Regulation (GDPR) and the Health Insurance Portability and Accountability Act (HIPAA), the confidentiality of these information should be guaranteed. Moreover, traditional coarse-grained access control mechanisms are unsuitable because they allow a user to get a whole PIIF with all-or-nothing entries, but cannot restrict the access rights of each entry. For example, the SSN in the PIIF may be only opened to the government and the bank while allergens are only allowed housekeeping attendants and doctors to access. Therefore, we require a flexible and fine-grained access control mechanism.

*Reliable user revocation.* When a large number of parties joining the retrieval service, how to revoke inactive or corrupt users stably and efficiently would be challenging. In particular, it requires that the user should be revoked immediately, even if a few involved servers collapse or they are compromised. Without such security guarantee, it would cause unpredictable data leakage since a revoked user may still be able to access some PIIFs. Thus, we demand for a reliable user revocation to further guarantee the data privacy.

*Data verification for multiple servers.* When a party requests a PIIF from the retrieval service, the received result may be processed by multiple servers. Once one of them produces a wrong result, it would cause serious consequences, such as giving a fatal prescription due to wrong allergens. Therefore, we need a verification mechanism to check the correctness of received results and locate the misbehaved server in the cluster to avoid accidents.

In this paper, we investigate these issues and try to give a working solution.

### Known techniques and their limitations

Attribute-Based Encryption (ABE) (Sahai and Waters 2005) is a promising solution to provide data privacy and flexible access control. Goyal et al. (2006) formalized two types of ABE: key-policy ABE (KP-ABE) (Ostrovsky et al. 2007; Okamoto and Takashima 2010; Lewko et al. 2010) and ciphertext-policy ABE (CP-ABE) (Bethencourt et al. 2007; Waters 2011) which is suitable for data sharing among multi-party. For simplicity, we limit the discussions to CP-ABE hereafter. A user is assigned a secret key with a number of attributes while data is encrypted by an access policy which is formed by attributes and a Boolean expression. Only when the user’s attributes satisfy the access policy in the ciphertext, it can decrypt. However, there are still a few subtle limitations.

- *Lacking efficient and reliable user revocation mechanism.* In the literature, traditional revocation mechanisms for ABE fall into two categories, namely indirect revocation (Attrapadung and Imai 2009; Cui et al. 2016; Qin et al. 2017), and direct revocation (Attrapadung and Imai 2009; Datta et al. 2016). However, these works suffer from limited scalability as either all ciphertexts should be updated or all user secret keys (or proxy-side keys) should be updated when revoking a user from system. Recently, server-aided approach (Yang et al. 2015; Ma et al. 2019) has been proposed to efficiently revoke a user from system. The server, which holds a cloud-side secret key and an authorized user list, performs the immediate user revocation by refusing to process the decryption requests of revoked users. However, it is weak for the cluster setting since multiple servers would hold a same cloud-side secret key. Once a server is compromised, the revocation mechanism would be broken.
- *Needing verification mechanism to locate a misbehaved cloud server for wrong results.* Outsourced decryption was proposed by Green et al. (2011) to improve the decryption efficiency and Lai et al. (2013) put forward a property called verifiability to check the correctness of outsourced decryption result. Later on, the works (Mao et al.^2015^; Ma et al. 2015; Lin et al. 2015) further improved the performance. More recently, Ge et al. (2021) proposed a method to verify the re-encryption result. However, all above verification mechanisms are inapplicable when multiple servers are deployed, where more cloud servers could make mistakes for one cooperative computation operation. They cannot locate a misbehaved server from the cluster when a wrong result is found.

Besides, the existing works (Goyal et al. 2006; Bethencourt et al. 2007; Waters 2011; Attrapadung and Imai 2009; Green et al. 2011; Lai et al. 2013; Yang et al. 2015; Ma et al. 2015; Ma et al. 2019; Ge et al. 2021) only benchmarked the performance of algorithms, but did not figure out how to integrate their cryptographic schemes in real-world system and give systematic solutions in practice.

### Our contributions

To tackle the above challenges, we design and implement *Rainbow*, a practical PII retrieval scheme which involves modern cloud techniques and cryptographic tools, including a well-designed ABE scheme called *Reliable Outsourced ABE* (ROABE). Some dedicated techniques of Rainbow are highlighted as follows:

*Field-level and fine-grained access control.* In Rainbow, fine-grained access control and data encryption are all done by ROABE. A PII owner can flexibly pose an access policy on every single entry (field) in a PIIF via ABE encryption, e.g., using policy “*All*” to encrypt the entry “Name: Alice” while using “*Government or Bank*” to encrypt the entry “SSN: XXX”. Only the user whose attribute set satisfies the access policy can recover the encrypted entry.

*Reliable immediate user revocation.* We design a reliable immediate user revocation mechanism with assistance of cloud servers. In particular, when a user is no longer involved, he will be revoked immediately by simple operations and cannot access any PIIF. Moreover, our user revocation mechanism is reliable since it can still work even if few cloud servers are compromised which leads to the leakage of cloud-side secret key.

*Verification mechanism for multiple servers.* We propose a verification mechanism to trace misbehaviors from multiple cloud servers. Users can efficiently verify the PIIF returned from servers. Once a wrong result is detected, the misbehaved cloud server will be identified via digital evidence and cannot exculpate itself.

*Systematic implementation with ownCloud.* We implement Rainbow based on own Cloud (https://owncloud.org/), a popular cloud storage hosting software, and use several industrial techniques, such as Message Queue, PKI, to deploy Rainbow in real world for providing PII retrieval service. The functionalities and performances are evaluated in mainstream cloud platforms, including AWS, GCP and Azure, and on PC (in browsers) & Android devices. Both the theoretical and experimental results show that Rainbow is practical.

### Technical overview

In this section, we briefly introduce our design ideas.

We borrow an idea from Yang et al. (2015) to achieve immediate user revocation, combining a cloud-side secret key, a user-side secret key and an original ABE secret key to form a proxy key. Then, the decryption requires the collaboration of both the cloud server and the user, and the user decryption capability can be immediately revoked if the cloud server refuses to help. Furthermore, to make user revocation mechanism more reliable, taking advantage of the architecture, we adopt (t, n) Shamir secret sharing to split the cloud-side secret key and each server maintains a unique share as its cloud-side secret key. The mechanism is reliable since it can tolerate at most ${t}-1$ keys to be compromised.

To guarantee the verifiability of all computation results from multiple servers within an outsourced decryption task to locate a misbehaved server, we follow the existing verifiable ABE schemes (Lai et al. 2013; Ma et al. 2015), which used the Pederson commitment (Pedersen 1991) to verify the final decryption result. Besides, we require more verifiable features since more servers are needed to help with outsourced decryption. In particular, the decryption shares, which are produced by t servers and used to implicitly recover the original cloud-side secret key, should be verified. We adopt Rabin’s technique (Rabin 1994) to give a private verification of the decryption share.

To initialize Rainbow in real world, first, similar to *WebCloud* (Sun et al. 2020), which was proposed by Sun et al. we utilize WebAssembly (W3C Community Group 2017), which is a low-level binary instruction format enabling deployment on the web and providing faster execution than JavaScript, to implement ROABE in browsers and adapt with ownCloud. Second, to provide cryptographic interfaces on ownCloud servers, we build the dynamic library of ROABE. Third, we use cross-compilation technique to build an Android-support library for mobile clients. Last but not least, many industrial techniques, such as Message Queue, PKI, are used to enhance the practicality of Rainbow in real world. Specifically, we used JSON, one of the most popular data-interchange formats, to form the PIIF.

Combining all the above techniques, we are then able to solve the issues that we discussed before and build a secure and practical PII retrieval scheme.

### Future prospects

We also give three promising application scenarios with Rainbow.

1. *Automated form filling.* An old people can upload his PIIF to Rainbow. When he wants to transact business with any third party, such as the bank, the government, this application can help him to fill their information quickly. With the access control that provided by Rainbow, only authorized entries in the form can be automatically filled with matched fields.
2. *Flexible single sign-on (SSO).* Rainbow can help with password management for different websites with distinct access policies since authentication credentials are part of PII. Moreover, for SSO, the system authentication token is encrypted and stored in Rainbow. The user whose attributes satisfy the access policy can recover the token and use it for authentication. The token should be refreshed once used.
3. *Secure data fusion.* Data fusion is an advanced technology to produce accurate information by integrating multiple data sources. Rainbow can protect the sensitive information in different data sources. In particular, before users delegating their data to the data fusion service, they could arbitrarily set the access policy of each field and encrypt it. Then only authorized fields would be fused. Besides, it is allowed to flexibly specify who is authorized to access the derived dataset resulting from the fusion for different usage.

## Preliminary

*Notations.* ${\textsf{Alg}}(\mathtt{arg_1},\mathtt{arg_2},\ldots ,\mathtt{arg_n})\rightarrow (\vartheta _1,ldots,\vartheta _m)$ denote running algorithm ${\textsf{Alg}}$ with input $\mathtt{arg_1}$, $\mathtt{arg_2}$,ldots, $\mathtt{arg_n}$ and obtaining outputs $\vartheta _1$,..., $\vartheta _m$. If ${\mathcal{S}}$ is a set, let $|{\mathcal{S}}|$ be its size. The symbol $s {\mathop {\leftarrow }\limits ^{\$}}{\mathcal{S}}$ means that an element *s* is randomly chosen from a set ${\mathcal{S}}$. The concatenation of two strings *x* and *y* is described by the symbol $x\Vert y$. A function *f* is negligible if for every $\kappa >0$, there exists $\lambda ' > 0$ such that $f(\lambda ) < 1/\lambda ^\kappa$ for all $\lambda > \lambda '$. Let $\Delta _{\delta ,{\mathcal{J}}}$ denote the Lagrange coefficient for $\delta \in {{\mathbb{Z}}_{p}}$ and a set ${\mathcal{J}}$ of elements in ${{\mathbb{Z}}_{p}}$: $\Delta _{\delta ,{\mathcal{J}}}(x)=\prod _{j\in {\mathcal{J}},j \ne \delta }\frac{x-j}{\delta -j}$. Let $[n]=\{1,2,\ldots ,n\}$ and $\Phi _z$ denote a set where $0< z\le n$, $|\Phi _z| = z$ and $\Phi _z \subseteq [n]$.

### Definition 1

(*Bilinear Maps*) Assume there exist two multiplicative cyclic groups ${\mathbb{G}}$ and ${\mathbb{G}}_{\text{T}}$ with a same prime order *p*.

A map $e:{\mathbb{G}}\times {\mathbb{G}}\rightarrow {\mathbb{G}}_{\text{T}}$ is called bilinear map if it is efficiently computable and has the following properties: 1) Bilinearity: $\forall$ $h_1,h_2\in {\mathbb{G}}$ and $\forall$ $a, b\in {\mathbb{Z}}_{p}$, $e(h_1^a, h_2^b)=e(h_1,h_2)^{a b}$. 2) Nondegeneracy: $e(h_1,h_2)\ne 1_{{\mathbb{G}}_{\text{T}}}$ if $h_1,h_2\ne 1_{{\mathbb{G}}}$.

### Definition 2

(*The Generic Bilinear Group Model*) The definition follows (Boneh et al. 2005). In generic bilinear group model, there are two random encodings over ${\mathbb{F}}_{p}$, which are injective maps, $\varphi : {\mathbb{F}}_{p}\rightarrow \{0,1\}^n$, $\varphi _{T}: {\mathbb{F}}_{p}\rightarrow \{0,1\}^n$, where ${\mathbb{F}}_{p}$ is the additive group and $n > 3log(p)$. Let ${\mathbb{G}}= \{\varphi (x): x\in {\mathbb{F}}_{p}\}$ and ${\mathbb{G}}_{\text{T}}= \{\varphi _{T}(x): x\in {\mathbb{F}}_{p}\}$. The oracles are given to execute the induced group computation on ${\mathbb{G}}, {\mathbb{G}}_{\text{T}}$ and a non-degenerate bilinear map $e: {\mathbb{G}}\times {\mathbb{G}}\rightarrow {\mathbb{G}}_{\text{T}}$. Then ${\mathbb{G}}$ is refered to be a generic bilinear group.

### Definition 3

(*Access Tree* (Goyal et al. 2006)) An access policy which is in the form of monotonic formula, e.g., $attr_1$ and $attr_2$ or $attr_3$, can be transformed to an *access tree*, where an attribute is related to a leaf node and a threshold gate is assigned to a non-leaf node. In particular, the attribute associated with leaf node *j* is described by the symbol $A(j)$. Let $\omega$ be a non-leaf node, $th_\omega$ be its threshold value where $0<th_\omega \le N_\omega$ and $N_\omega$ be the number of its child nodes. It is obvious that the $\omega$ is an OR gate if $th_\omega =1$ and it is an AND gate if $th_\omega =N_\omega$. We also define the parent of $\omega$ using a symbol $pt(\omega )$. Besides, each node of the tree is ordered and the function $idx(\omega )$ produces a unique number associated with the order of $\omega$, e.g., suppose the tree contains *n* nodes and the inorder traversal of these nodes is $\omega _1,\omega _2,\ldots \omega _n$, the function $idx(\omega _i)$ could output *i* as the unique number of $\omega _i$.

### Definition 4

(*Satisfying an Access Tree* (Goyal et al. 2006)) Let $\mathcal{T}$ be an access tree and $\mathcal{T}_\omega$ be the subtree of $\mathcal{T}$ rooted at node $\omega$. We use a binary relation *Q* to define the relationship between a attribute set and an access tree. In particular, let ${\mathcal{R}}$ denote an attribute set, when $\omega$ is a non-leaf node, $Q(\mathcal{T}_\omega ,{\mathcal{R}})$ is computed recursively as follows: it computes $Q(\mathcal{T}_{\omega _c},{\mathcal{R}})$ for all child nodes $\omega _c$ of $\omega$. $Q(\mathcal{T}_\omega ,{\mathcal{R}})$ returns 1 if and only if as least $th_\omega$ child nodes return 1.

When $\omega$ is a leaf node, $Q(\mathcal{T}_\omega ,{\mathcal{R}})$ returns 1 if and only if the attribute is matched, in other words, $A(\omega )\in {\mathcal{R}}$. If none of the above cases is satisfied, $Q(\mathcal{T}_\omega ,{\mathcal{R}})=0$.

## Overview of rainbow

In this section, we present the system model and the design goals of Rainbow. Some useful acronyms are summarized in Table 1.

**Table 1:** Acronyms used in this paper

Acronym	Description	Acronym	Description
TA	Trusted authority	CSP	cloud service provider
PO	PII owner	PU	PII user
MCS	Master cloud server	HCS	helping cloud server
$\texttt{pk}$	Public key	$\texttt{msk}$	master secret key
$\texttt{cpk}$	Cloud-side public key	${\texttt{csk}}_{i}$	the *i*th cloud-side secret key
$\texttt{upk}$	User public key	$\texttt{usk}$	user secret key
$\texttt{dk}$	Delegated key	${\mathcal{L}}$	delegated key list
${\texttt{ds}}_{j,i}$	The decryption share generated by the *j*th server for the *i*th server	${\pi }_{j,i}$	the proof of $\texttt{ds}_{j,i}$
$\texttt{ct}$	Ciphertext	$\texttt{dct}$	partially decrypted ciphertext
$\texttt{csv}$	Combined secret value		

### System model

**Fig. 1:**
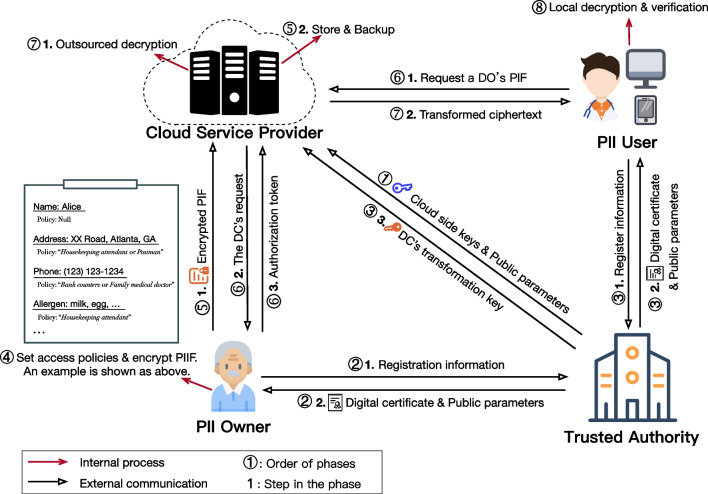
System model of Rainbow

As shown in Fig. 1, four entities are involved in Rainbow: Trusted Authority (TA), Cloud Service Provider (CSP), PII Owner (PO) and PII User (PU). Each entity is explained as follows.

*TA* is an honest entity. It is assigned to process sensitive information, including initializing system (see Phase ${\textcircled{\scriptsize{1}}}$) and generating digital certificates and delegated keys for users (see Phase ${\textcircled{\scriptsize{2}}}$ & ${\textcircled {\scriptsize{3}}}$). In particular, it generates systematic public parameters and several cloud side keys for system warming-up. The delegated key is used to decrypt the encrypted PIIF.

*CSP* consists of multiple servers over multi-cloud and the following four services.

- *Upload service* processes upload requests from PII owners and provides reliable storage (see Phase ${\textcircled {\scriptsize{5}}}$, Step 2). Once a PIIF is uploaded to CSP, it will be stored and made a backup by this service. Note that all PIIFs are encrypted.
- *Confirmation service* processes download requests from PII users. When a PII user requests to access a PIIF, this service transfers the request to the PII owner for confirmation (see Phase ${\textcircled {\scriptsize{6}}}$, Step 2). Besides, it checks the response from the PII owner. Only when the response indicates that the PII user is allowed to obtain the PIIF, CSP would provide outsourced decryption with his delegated key.
- *Outsourced decryption service* decrypts encrypted entries in the requested PIIF to transformed ciphertexts using the PII user’s delegated key and returns them to the PII user (see Phase ${\textcircled {\scriptsize{7}}}$, Step 1 & 2). An outsourced decryption task involves several servers and the computation results of each server can be verified. Besides, without this service, nobody can decrypt a ciphertext.
- *User revocation service* maintains a delegated key list associated with users for revocation. It revokes an inactive user by removing the corresponding entry from the list. By the way, once the user is revoked, the Outsourced Decryption Service refuses to help with the decryption.

*PO* decides a subset of his PII that to be shared and forms a specific PIIF. Then he encrypts the PIIF and uploads it to CSP (see Phase ${\textcircled {\scriptsize{4}}}$ & Phase ${\textcircled {\scriptsize{5}}}$, Step 1). In particular, he can set any desired access policy (formed by attributes and Boolean expressions) of each entry in the PIIF. For example, as shown in Fig. 1, the entry “Address” is encrypted by the policy “*Housekeeping attendant or Postman*” while “Allergern” is encrypted by the policy “*Housekeeping attendant*”. Moreover, the PO generates a confirmation token (or a rejection token) to CSP when a user requests to access the PO’s PIIF (see Phase ${\textcircled {\scriptsize{6}}}$, Step 3).

*PU* consumes the encrypted PIIF from CSP, e.g., it can be a doctor, a bank staff and a housekeeping attendant. The access rights of PUs are described by a number of attributes. A PU gets a secret key and a corresponding delegated key which is associated with an attribute set from TA when he registers to the system (see Phase ${\textcircled {\scriptsize{3}}}$). If his attribute set satisfies the access policy of an entry in a PIIF, he can decrypt the encrypted entry and check the correctness (see Phase ${\textcircled {\scriptsize{8}}}$).

We assume that TA and PO are honest. Most of cloud servers in CSP are assumed to be honest, while few of them are assumed to be “covert” adversaries who may deviate from the outsourced decryption protocol and try to produce unsatisfied decryption results, but are unwilling to be caught. As for PUs, we assume that a majority of them are honest, while few of them are corrupt and leakage their secret keys in the collision to access unauthorized data. This mimics the real world since some devices of PUs may be lost and be corrupted by a few spiteful people.

### Design goals

Based on aforementioned system model and trust assumptions, Rainbow should meet the following design goals.

*Data privacy.* Due to the sensitive personal information (e.g., address, phone number, social security number) involved in a PIIF, any PIIF that is sent and outsourced to public clouds should be only accessed by authorized users.

*Mandatory and flexible access control.* The PO should be able to arbitrarily decide which entries in his PIIF need encryption and the access policy of each entry. Nobody can recover the information of these encrypted entries if his attribute set does not satisfies the policies, even he can obtain all these ciphertexts.

*Efficient and reliable user revocation.* Once a PU becomes inactive, he should be revoked with low costs. Moreover, since there are multiple servers in Rainbow to provide services, we require that even few servers are compromised to work for a revoked user, he cannot obtain any useful information of the encrypted PIIFs.

*Full verifiability.* In Rainbow, we need a feasible verification mechanism for PUs to check the outsourced decryption result. Furthermore, the verification should support to locate a misbehaved server if the result is wrong, because several servers are involved in an outsourced decryption task.

## An important tool: ROABE

Towards the design goals of Rainbow, we propose an important tool called *Reliable Outsourced ABE* (ROABE) and introduce it in this section.

### Overview

**Fig. 2:**
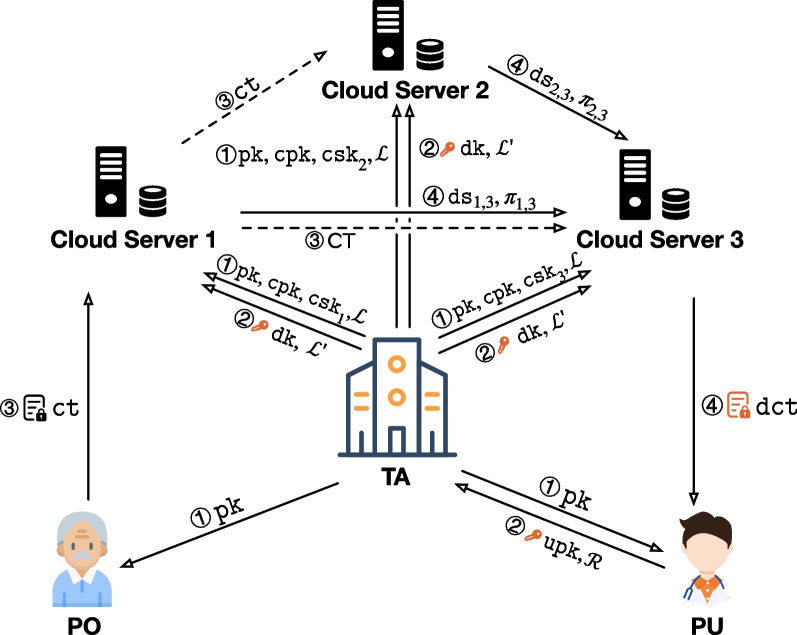
Model of ROABE

The model of ROABE is shown in Fig. 2 where multiple cloud servers are settled. An ROABE scheme consists of following 10 algorithms:

- ${\textsf{Setup}}(\lambda , {n}, {t})\rightarrow ({\texttt{pk}}, {\texttt{msk}}, {\texttt{cpk}}, \{{\texttt{csk}}_{i}\}_{i\in [n]}, {\mathcal{L}})$. On input a security parameter $\lambda$, the number of cloud servers n and a threshold t, it outputs a public key $\texttt{pk}$, a master secret key $\texttt{msk}$, a cloud-side public key $\texttt{cpk}$, a cloud-side secret key set $\{{\texttt{csk}}_{i}\}_{i\in [n]}$ and a delegated key list ${\mathcal{L}}$.
- ${\textsf{UKeyGen}}(\texttt{pk}, {u})\rightarrow (\texttt{upk},\texttt{usk})$. On input a public key $\texttt{pk}$ and an identity u, it outputs a user public key $\texttt{upk}$ and a user secret key $\texttt{usk}$.
- ${\textsf{DKeyGen}}({\texttt{msk}}, \texttt{cpk}, \texttt{upk}, {\mathcal{R}}, {\mathcal{L}})\rightarrow (\texttt{dk}, {\mathcal{L}}')$. On input a master secret key $\texttt{msk}$, a cloud-side public key $\texttt{cpk}$, a user public key $\texttt{upk}$, an attribute set ${\mathcal{R}}$ and a delegated key list ${\mathcal{L}}$, it outputs a delegated key $\texttt{dk}$ and an updated list ${\mathcal{L}}'$.
- ${\textsf{Encrypt}}(\texttt{pk}, \mathcal{T}, \texttt{m})\rightarrow \texttt{ct}$. On input a $\texttt{pk}$, an access tree $\mathcal{T}$ and a message $\texttt{m}$, it outputs a ciphertext $\texttt{ct}$.
- ${\textsf{DSGen}}({\texttt{csk}}_{j}, {\texttt{dk}}, i, {\texttt{ct}})\rightarrow ({\texttt{ds}}_{j,i}, {\pi }_{j,i})/\bot$. On input a cloud-side secret key ${\texttt{csk}}_{j}$ with the serial number *j*, a delegated key $\texttt{dk}$, a serial number *i* and a ciphertext $\texttt{ct}$, it outputs a decryption share ${\texttt{ds}}_{j,i}$ and a corresponding proof ${\pi }_{j,i}$ or $\bot$.
- ${\textsf{DSVerify}}({\texttt{csk}}_{i}, {\texttt{dk}}, {\texttt{ds}}_{j,i}, {\pi }_{j,i}, \texttt{ct})\rightarrow \texttt{b}$. On input a $\texttt{csk}_{i}$, a $\texttt{dk}$, a decryption share ${\texttt{ds}}_{j,i}$, a proof ${\pi }_{j,i}$ and a $\texttt{ct}$, it outputs a bit $\texttt{b} \in \{0,1\}$ where $\texttt{b}=1$ indicates that ${\texttt{ds}}_{j,i}$ is correct.
- ${\textsf{DSCombine}}({\texttt{cpk}}, \{{\texttt{ds}}_{j,i}\}_{j\in {\Phi }_{t}})\rightarrow \texttt{csv}$. On input a cloud-side public key $\texttt{cpk}$ and t decryption shares $\{{\texttt{ds}}_{j,i}\}_{j\in {\Phi }_{t}}$ where ${\Phi }_{t}\subseteq [n]$ and $|{\Phi }_{t}|=t$, it outputs a combined secret value $\texttt{csv}$.
- ${\textsf{CSDecrypt}}(\texttt{pk}, \texttt{dk}, \texttt{ct}, \texttt{csv})\rightarrow \texttt{dct}/\bot$. On input a $\texttt{pk}$, a $\texttt{dk}$, a $\texttt{ct}$ and a $\texttt{csv}$, it outputs a partially decrypted ciphertext $\texttt{dct}$ or $\bot$.
- ${\textsf{UDecrypt}}({\texttt{pk}, \texttt{dct}}, \texttt{usk})\rightarrow \mathtt{m'}/\bot$. On input a $\texttt{pk}$, a partially decrypted ciphertext $\texttt{dct}$ and a user secret key $\texttt{usk}$, it outputs a message $\mathtt{m'}$ or $\bot$.
- $\textsf{URevoke}({u},{\mathcal{L}})\rightarrow {\mathcal{L}}'$. On input a u and a ${\mathcal{L}}$, it outputs an updated list ${\mathcal{L}}'$.

The algorithms Setup, UKeyGen, DKeyGen, and Encrypt are probabilistic and DSGen, DSVerify, DSCombine, CSDecrypt, UDecrypt, and URevoke are deterministic. The keys $\texttt{msk}$, $\texttt{cpk}$, ${\texttt{csk}}_{i}$ ($\forall i\in [n]$) contain $\texttt{pk}$.

*Correctness* The ROABE scheme is correct for all attribute sets ${\mathcal{R}}$, all access trees $\mathcal{T}$ where ${\mathcal{R}}$ satisfies $\mathcal{T}$, all $({\texttt{pk}}, {\texttt{msk}}, {\texttt{cpk}}, \{{\texttt{csk}}_{i}\}_{i\in [n]}, {\mathcal{L}})\in {\textsf{Setup}}(\lambda , {n}, {t})$ where ${t}\le {n}$, all $(\texttt{upk}, \texttt{usk})\in {\textsf{UKeyGen}}(\texttt{pk}, {u})$, all $\texttt{dk}\in {\textsf{DKeyGen}}(\texttt{msk}, \texttt{cpk}, \texttt{upk}, {\mathcal{R}}, {\mathcal{L}})$, all $\texttt{ct} \in {\textsf{Encrypt}}(\texttt{pk}, \mathcal{T}, \texttt{m})$, all ${({\texttt{ds}}_{j,i}, {\pi }_{j,i})}\in {\textsf{DSGen}}({\texttt{csk}}_{j}, {\texttt{dk}}, i, {\texttt{ct}})$ where $i,j\in [n], j\ne i$, all ${\texttt{csv}}\in {\textsf{DSCombine}}({\texttt{cpk}}, \{{\texttt{ds}}_{j,i}\}_{j\in {\Phi }_{t}})$ where ${\Phi }_{t}\subseteq [n]$ and $|{\Phi }_{t}|= {t}$, all $\texttt{dct}\in {\textsf{CSDecrypt}}(\texttt{pk}, \texttt{dk}, \texttt{ct}, \texttt{csv})$, and all $\mathtt{m'}\in {\textsf{UDecrypt}}({\texttt{pk}, \texttt{dct}}, \texttt{usk})$, if $\mathtt{m'}\ne \bot$, $\mathtt{m'}=\texttt{m}$ and ${\textsf{DSVerify}}({\texttt{csk}}_{i}, {\texttt{dk}}, {\texttt{ds}}_{j,i}, {\pi }_{j,i}, \texttt{ct})=1$.

We now describe the workflow of ROABE. It contains six phases, including system initialization (see ${\textcircled {\scriptsize{1}}}$ in Fig. 2), user registration (see ${\textcircled {\scriptsize{2}}}$), data upload (see ${\textcircled {\scriptsize{3}}}$), data download (see ${\textcircled {\scriptsize{4}}}$), local decryption and user revocation. As shown in Fig. 2, take ${n}=3$, ${t}=2$ for example where three cloud servers are settled and the threshold value is two, at least two cloud servers are needed to complete the outsourced decryption. We also mark each cloud server with a serial number, e.g., the serial number of Cloud Server 1 is *1*. More details of the model are discussed as follows and some useful acronyms are listed in Table 1.

1. *System initialization.* TA runs the algorithm ${\textsf{Setup}}$ to generate a public key $\texttt{pk}$, a master secret key $\texttt{msk}$, a cloud-side public key $\texttt{cpk}$, a set of cloud-side secret key $\{{\texttt{csk}}_{i}\}_{i\in [n]}$ and an empty delegated key list ${\mathcal{L}}$. The secret key ${\texttt{csk}}_{i}$ is securely transmitted to the *i*th cloud server along with ($\texttt{pk}$, $\texttt{cpk}$, ${\mathcal{L}}$) and $\texttt{pk}$ is published.
2. *User registration.* A PU runs $${\textsf{UKeyGen}}$$$$(\texttt{pk}, {u})$$ where u is his identity to obtain a user public key $$\texttt{upk}$$ and a user secret key $$\texttt{usk}$$. Then the PU sends his attribute set $${\mathcal{R}}$$ along with $$\texttt{upk}$$ to TA. TA runs $${\textsf{DKeyGen}}$$$$(\texttt{msk}, \texttt{cpk}, \texttt{upk}, {\mathcal{R}}, {\mathcal{L}})$$ to generate a delegated key $$\texttt{dk}$$ associated with $${\mathcal{R}}$$ and an updated list $${\mathcal{L}}'$$. Note that an entry in $${\mathcal{L}}$$ is $$({u}, \texttt{dk})$$. The key $$\texttt{dk}$$ and the updated list $${\mathcal{L}}'$$ are sent to all servers.
3. *Data upload.* A PO runs $${\textsf{Encrypt}}$$$$(\texttt{pk}, \mathcal{T}, \texttt{m})$$ to encrypt the message $$\texttt{m}$$ with the access tree $$\mathcal{T}$$ to get a ciphertext $$\texttt{ct}$$. Then he uploads the ciphertext $$\texttt{ct}$$ to a cloud server. The server synchronizes $$\texttt{ct}$$ to others to make a backup.
4. *Data download.* When a PU u wants to download a $$\texttt{ct}$$ from a cloud server, called master cloud server (MCS), the MCS sends an assistance request of $$\texttt{ct}$$ to other t cloud servers, called helping cloud server(s) (HCS), for help, if the entry (u, $$\texttt{dk}$$) is in $${\mathcal{L}}$$. Otherwise, it rejects to provide service. For example, in Fig. 2, Cloud Server 1 & 2 are the HCSs and Cloud Server 3 is the MCS in this session. Let *i* be the serial number of MCS. The HCS whose serial number is *j* rejects to provide service if (u, $$\texttt{dk}$$) is not in its list $${\mathcal{L}}$$. Otherwise, it runs $${\textsf{DSGen}}$$$$({\texttt{csk}}_{j}, {\texttt{dk}}, i, \texttt{ct})$$ to get a decryption share $${\texttt{ds}}_{j,i}$$ and a corresponding proof $${\pi }_{j,i}$$ and returns $$({\texttt{ds}}_{j,i}, {\pi }_{j,i})$$ to the MCS. Or $$\bot$$ is output, the HCS returns a response message “Unsatisfied attributes” to indicate that the attributes of the PU cannot satisfy the access tree of $$\texttt{ct}$$. Then the MCS runs $${\textsf{DSVerify}}$$$$({\texttt{csk}}_{i}, {\texttt{dk}}, {\texttt{ds}}_{j,i}, {\pi }_{j,i}, \texttt{ct})$$ to check the validity of $${\texttt{ds}}_{j,i}$$. All other $${t}-1$$ decryption shares are also verified. Only when at least t valid decryption shares are obtained, the MCS runs $${\textsf{DSCombine}}$$$$({\texttt{cpk}},\{{\texttt{ds}}_{j,i}\}_{j\in {\Phi }_{t}})$$ to get a combined secret value $$\texttt{csv}$$ and runs $${\textsf{CSDecrypt}}$$$$(\texttt{pk}, \texttt{dk}, \texttt{ct}, \texttt{csv})$$ to get a partially decrypted ciphertext $$\texttt{dct}$$ and returns it to the user.
5. *Local decryption.* When a PU receives $$\texttt{dct}$$ from a cloud server, he runs $${\textsf{UDecrypt}}$$$$(\texttt{pk}, \texttt{dct}, \texttt{usk})$$ to obtain a recovered message $$\mathtt{m'}$$ or $$\bot$$. Note that only when $$\texttt{dct}$$ is correctly generated by the $$\texttt{dk}$$ which is produced by the $$\texttt{upk}$$ corresponding to $$\texttt{usk}$$, a correct message can be output. Hence, it can verify the correctness of $$\texttt{dct}$$.
6. *User revocation.* Once a PU u is suggested to be revoked, all cloud servers should run $${\textsf{URevoke}}$$$$({u}, {\mathcal{L}})$$ to remove the entry (u,$$\texttt{dk}$$) from $${\mathcal{L}}$$. Then the cloud servers do not provide outsourced decryption for u. Without the help of cloud servers, u cannot decrypt ciphertexts anymore.

### Security threats and formal definitions

In this section, we discuss the security threats and give the formal security definitions of ROABE.

Three aspects of security are considered for ROABE, namely, data privacy, reliable user revocation and full verifiability. Data privacy requires that unauthorized users and clouds are ignorant of encrypted data. Reliable user revocation demands that once a PU is revoked, it cannot decrypt any $\texttt{ct}$, even a few (less than a threshold t) servers still provide outsourced decryption service for it. Full verifiability requires that all outsourced decryption results, e.g., decryption shares $\texttt{ds}$ and partially decrypted ciphertexts $\texttt{dct}$, should be verified to locate a misbehaved server.

According to the involved entities and their possible behaviors, we consider five types of adversaries which threat the above requirements as follows. In particular, we assume that the public keys ($\texttt{pk}$, $\texttt{cpk}$) are held by all adversaries.

i. *Type-1 adversary* refers to some corrupted users attempting to collude together to decrypt unauthorized $\texttt{ct}$ to break the data privacy against users. It can obtain corrupted users’ delegated keys $\texttt{dk}$ and secret keys $\texttt{usk}$ and all ciphertexts $\texttt{ct}$. Moreover, we allow it to obtain all cloud-side secret keys $\{{\texttt{csk}}_{i}\}_{i\in [n]}$.
ii. *Type-2 adversary* refers to some cloud servers attempting to decrypt a $\texttt{ct}$ to break the data privacy against clouds. It can obtain all delegated keys $\texttt{dk}$, a few user secret keys $\texttt{usk}$ which are not associated with $\texttt{dk}$, all cloud-side secret keys $\{{\texttt{csk}}_{i}\}_{i\in [n]}$ and all $\texttt{ct}$. Note that it cannot hold any key pair ($\texttt{dk}$, $\texttt{usk}$) to trivially decrypt a $\texttt{ct}$.
iii. *Type-3 adversary* refers to a revoked user trying to decrypt a $\texttt{ct}$ to break the reliable user revocation. It can obtain all $\texttt{dk}$, all $\texttt{usk}$, ${t}-1$ cloud-side secret keys $\{{\texttt{csk}}_{i}\}_{i\in {\Phi }_{t-1}}$ and all ciphertexts $\texttt{ct}$.
iv. *Type-4 adversary* refers to an HCS attempting to generate a wrong decryption share to pass the verification from an MCS (indexed by *i*) to break the verifiability of decryption share. It can obtain all $\texttt{dk}$, all $\texttt{usk}$, all cloud-side secret keys except the *i*th one $\{{\texttt{csk}}_{k}\}_{k\in [n],k\ne i}$ and all $\texttt{ct}$.
v. *Type-5 adversary* refers to an MCS trying to generate a wrong partially decrypted ciphertext to pass the verification from a PU to break the verifiability of partially decrypted ciphertext. It can obtain all $\texttt{dk}$, all $\texttt{usk}$, all $\{{\texttt{csk}}_{i}\}_{i\in [n]}$ and all $\texttt{ct}$.

We now give the formal security definitions as follows. Let $\mathcal{C}$ be the challenger and $\mathcal{A}$ be the adversary.

We follow the security definition of data privacy against users in Yang et al. (2015) which used indistinguishability against chosen plaintext attack (IND-CPA) model, to define the data privacy against users of ROABE.

#### Definition 5

(*Data Privacy against Users*) An ROABE scheme achieves data privacy against users if any probabilistic polynomial time (PPT) type-1 adversary has at most a negligible advantage to win the following security game $\texttt{Game}_{\text{priv}}^{\text{du}}$.

${{\textsc {Setup}}}$ $\mathcal{C}$ runs ${\textsf{Setup}}$ and returns $({\texttt{pk}}, {\texttt{cpk}}, \{{\texttt{csk}}_{i}\}_{i\in [n]}, {\mathcal{L}})$ to $\mathcal{A}$. $\mathcal{C}$ also initializes an empty table $\mathcal{W}$.

${{\textsc {Phase 1}}}$ $\mathcal{A}$ is allowed to query following oracles:

- *User-key oracle* $$\mathcal{O}_{\textit{UK}}(u)$$: $$\mathcal{C}$$ runs $${\textsf{UKeyGen}}$$$$(\texttt{pk}, u)$$ to return $$(\texttt{upk},\texttt{usk})$$ to $$\mathcal{A}$$ and stores $$({u}, \texttt{upk}, \texttt{usk})$$ to $$\mathcal{W}$$.
- *Delegated-key oracle* $$\mathcal{O}_{\textit{DK}}({\mathcal{R}}, u)$$: $$\mathcal{C}$$ gets the $$\texttt{upk}$$ from $$\mathcal{W}$$ that is indexed by u and rejects if no such $$\texttt{upk}$$ exists. Otherwise, with the queried attribute set $${\mathcal{R}}$$, $$\mathcal{C}$$ runs $${\textsf{DKeyGen}}$$$$(\texttt{msk}, \texttt{cpk}, \texttt{upk}, {\mathcal{R}}, {\mathcal{L}})$$ to get a $$\texttt{dk}$$ and returns it to $$\mathcal{A}$$. Note that $$\mathcal{A}$$ can add $$({u}, \texttt{dk})$$ to $${\mathcal{L}}$$ by himself.

*Challenge* $\mathcal{A}$ submits two message $\texttt{m}_{0}, \texttt{m}_{1}$ where $|\texttt{m}_{0}|=|\texttt{m}_{1}|$ and an access tree ${\mathcal{T}}^{*}$, subjecting to a restriction that none of the queried attribute set ${\mathcal{R}}$ in Phase 1 satisfies ${\mathcal{T}}^{*}$. $\mathcal{C}$ flips a random coin *b* and runs Encrypt $(\texttt{pk}, \mathcal{T^*}, \texttt{m}_{b})$ to obtain $\texttt{ct}^*$. Finally, $\mathcal{C}$ returns $\texttt{ct}^*$ to $\mathcal{A}$.

*Phase 2* $\mathcal{A}$ continues to query the oracles with the restriction that any queried ${\mathcal{R}}$ does not satisfy ${\mathcal{T}}^{*}$.

*Guess* $\mathcal{A}$ outputs a guess $b'$ of *b*. $\mathcal{A}$ wins the game if $b=b'$.

Similarly, we follow the security definition of data privacy against cloud server in Yang et al. (2015) to define the data privacy against clouds of ROABE. In particular, the adversary is not allowed to obtain a key pair $(\texttt{dk}, \texttt{usk})$ to trivially decrypt the challenge ciphertext.

#### Definition 6

(*Data Privacy against Clouds*) An ROABE scheme achieves data privacy against clouds if any PPT type-2 adversary has at most a negligible advantage to win the following security game $\texttt{Game}_{\text{priv}}^{\text{cs}}$.

${{\textsc {Setup.}}}$ Same as Setup in $\texttt{Game}_{\text{priv}}^{\text{du}}$.

${{\textsc {Phase 1.}}}$ $\mathcal{A}$ is allowed to query following oracles:

- *User-key oracle* $\mathcal{O}_{\textit{UK}}(u)$: $\mathcal{C}$ runs ${\textsf{UKeyGen}}$ to get $(\texttt{upk}, \texttt{usk})$, stores $({u}, \texttt{upk}, \texttt{usk})$ to $\mathcal{W}$ and returns $\texttt{upk}$ to $\mathcal{A}$.
- *Delegated-key oracle* $$\mathcal{O}_{\textit{DK}}({\mathcal{R}}, u)$$: $$\mathcal{C}$$ rejects if the entry $$({u},\texttt{upk},\texttt{usk})$$ is in $$\mathcal{W}$$. Otherwise, $$\mathcal{C}$$ runs $${\textsf{UKeyGen}}$$$$(\texttt{pk}, u)$$ and $${\textsf{DKeyGen}}$$$$(\texttt{msk}, \texttt{cpk}, \texttt{upk}, {\mathcal{R}}, {\mathcal{L}})$$ to get $$(\texttt{upk}, \texttt{usk})$$ and $$\texttt{dk}$$. Finally, $$\mathcal{C}$$ returns $$(\texttt{dk}, \texttt{upk})$$ to $$\mathcal{A}$$.

${{\textsc {Challenge.}}}$ Almost same as Challenge in $\texttt{Game}_{\text{priv}}^{\text{du}}$, except that the restriction of $\mathcal{T}^*$ is removed.

${{\textsc {Phase 2.}}}$ Same as Phase 1.

${{\textsc {Guess.}}}$ Same as Guess in $\texttt{Game}_{\text{priv}}^{\text{du}}$.

To define reliable user revocation, we follow the user revocation support in Yang et al. (2015). For a revoked user, it cannot decrypt any $\texttt{ct}$ even all keys and a few cloud-side secret keys are given to it. In particular, $t-1$ cloud-side secret keys are exposed to the adversary.

#### Definition 7

(*Reliable User Revocation*) An ROABE scheme achieves reliable user revocation if any PPT type-3 adversary has at most a negligible advantage to win the following indistinguishability game $\texttt{Game}_{\text{rvk}}^{\text{}}$.

${{\textsc {Setup.}}}$ Almost same as Setup in $\texttt{Game}_{\text{priv}}^{\text{du}}$, except that $\{{\texttt{csk}}_{i}\}_{i\in {\Phi }_{t-1}}$ is sent to $\mathcal{A}$ rather than $\{{\texttt{csk}}_{i}\}_{i\in [n]}$.

${{\textsc {Phase 1.}}}$ Same as Phase 1 in $\texttt{Game}_{\text{priv}}^{\text{du}}$.

${{\textsc {Challenge.}}}$ Almost same as Challenge in $\texttt{Game}_{\text{priv}}^{\text{du}}$, except that the restriction of $\mathcal{T}^*$ is removed.

${{\textsc {Phase 2.}}}$ Same as Phase 1.

${{\textsc {Guess.}}}$ Same as Guess in $\texttt{Game}_{\text{priv}}^{\text{du}}$.

To describe the verifiability of decryption share, similar to the verifiability defined in Lai et al. (2013), the adversary should produce two different tuples $({\texttt{ds}}_{j,i},{\pi }_{j,i})$ and $({\texttt{ds}}_{j,i}',{\pi }_{j,i}')$ where one of them is incorrect. Note that the adversary can always compute a correct decryption share by runnning DSGen with a corrupt ${\texttt{csk}}$. Besides, if the adversary obtains the ${\texttt{csk}}$ of the *i*th cloud which it wants to cheat, it can trivially generate a wrong pair $({\texttt{ds}}_{j,i},{\pi }_{j,i})$ to pass the verification. Thus, we have that nobody can produce a wrong decryption share to pass the verification without a target ${\texttt{csk}}_{i}$.

#### Definition 8

(*Verifiability of Decryption Share*) An ROABE scheme achieves verifiability of decryption share if any PPT type-4 adversary has at most a negligible probability to win the following security game $\texttt{Game}_{\text{vrfy}}^{\text{ds}}$.

${{\textsc {Setup}}}$ $\mathcal{A}$ chooses a serial number $i^*$ as its attack target and sends $i^*$ to $\mathcal{C}$. $\mathcal{C}$ runs Setup with $({t},n)$ and returns $\texttt{pk}$, $\texttt{msk}$, $\texttt{cpk}$, $\{{\texttt{csk}}_{i}\}_{i\ne i^*,i\in [n]}$, ${\mathcal{L}}$ to $\mathcal{A}$.

${{\textsc {Phase 1.}}}$ Same as Phase 1 in $\texttt{Game}_{\text{priv}}^{\text{cs}}$.

${{\textsc {Challenge.}}}$ $\mathcal{A}$ submits a message $\texttt{m}^*$ and an access tree ${\mathcal{T}}^{*}$ to $\mathcal{C}$. $\mathcal{C}$ runs Encrypt $(\texttt{pk}, \mathcal{T^*}, \texttt{m}^*)$ to return ${\texttt{ct}^*}$ to $\mathcal{A}$.

${{\textsc {Phase 2.}}}$ Same as Phase 1.

${{\textsc {Output.}}}$ $\mathcal{A}$ outputs {$\texttt{dk}^{* }$, $({\texttt{ds}}^*_{j,i^*}, {\pi }^{*}_{j,i^*})$, $({\texttt{ds}}^{*\prime }_{j,i^*},{\pi }^{*\prime }_{j,i^*})$} where the attribute set ${\mathcal{R}}^*$ that is associated with $\texttt{dk}^{* }$ satisfies the challenge access tree in $\texttt{ct}^*$, and ${\texttt{ds}}^*_{j, i^*}\ne {\texttt{ds}}^{*\prime }_{j, i^*}$. Assume $\texttt{dk}^{* }$ has been generated by $\mathcal{O}_{\textit{DK}}$ and sent to $\mathcal{A}$ in Phase 1 or Phase 2. $\mathcal{A}$ wins the game if

$$\begin{aligned} &1\leftarrow {\textsf{DSVerify}}({\texttt{csk}}_{i^*}, {\texttt{dk}}^{* }, {\texttt{ds}}^*_{j,i^*}, {\pi }^*_{j,i^*}, {\texttt{ct}^*})\ \wedge \\&1\leftarrow {\textsf{DSVerify}}({\texttt{csk}}_{i^*}, {\texttt{dk}}^{* }, {\texttt{ds}}^{*\prime }_{j,i^*}, {\pi }^{*\prime }_{j,i^*}, {\texttt{ct}^*}). \end{aligned}$$

To define the verifiability of partially decrypted ciphertext, we follow the verifiability in Lai et al. (2013) that nobody can produce an incorrect $\texttt{dct}$ that can be decrypted as a valid message.

#### Definition 9

(*Verifiability of Partially Decrypted Ciphertext*) An ROABE scheme achieves verifiability of partially decrypted ciphertext if any PPT type-5 adversary has at most a negligible probability to win the following game $\texttt{Game}_{\text{vrfy}}^{\text{dct}}$.

${{\textsc {Setup.}}}$ Almost same as Setup in $\texttt{Game}_{\text{vrfy}}^{\text{ds}}$, except that $\mathcal{C}$ returns $\{{\texttt{csk}}_{i}\}_{i\in [n]}$ to $\mathcal{A}$.

${{\textsc {Phase 1.}}}$ Same as Phase 1 in $\texttt{Game}_{\text{vrfy}}^{\text{ds}}$.

${{\textsc {Challenge.}}}$ Same as Challenge in $\texttt{Game}_{\text{vrfy}}^{\text{ds}}$.

${{\textsc {Phase 2.}}}$ Same as Phase 1.

${{\textsc {Output.}}}$ $\mathcal{A}$ outputs a tuple {$\texttt{ct}^*, \texttt{usk}^{* }, {\texttt{dct}_{1}^{*}}, {\texttt{dct}_{2}^{*}}$}, where $\texttt{ct}^*$ is the challenge ciphertext produced by $\mathcal{C}$ in Challenge phase. Assume $\texttt{usk}^{* }$ has been hold by $\mathcal{A}$ in Phase 1 or Phase 2. Then $\mathcal{C}$ runs UDecrypt with $\texttt{usk}^{* }$ to decrypt ${\texttt{dct}_{1}^{*}}$ and ${\texttt{dct}_{2}^{*}}$ to get ${\texttt{m}_{1}^{*}}$ and ${\texttt{m}_{2}^{*}}$, respectively. $\mathcal{A}$ wins the game if ${\texttt{m}_{1}^{*}}\ne {\texttt{m}_{2}^{*}} \wedge {\texttt{m}_{1}^{*}}\ne \bot \wedge {\texttt{m}_{2}^{*}}\ne \bot$.

### A concrete construction

To initialize ROABE, in particular, we use a symmetric key encryption scheme SKE and a key derivation function ${\textsf{KDF}}$ (Krawczyk 2010) as building blocks. Specifically, we briefly review the definition of SKE.

A symmetric key encryption scheme SKE is a tuple of algorithms (${\textsf{Gen}}$, ${\textsf{Enc}}$, ${\textsf{Dec}}$) along with an associated key space ${\mathcal{K}}$, where:

- ${\textsf{Gen}}(1^\lambda )\rightarrow \kappa$. On input a security parameter $1^\lambda$, it outputs a key $\kappa \in {\mathcal{K}}$ where $|{\mathcal{K}}|\ge \lambda$.
- ${\textsf{Enc}}(\kappa , \texttt{msg})\rightarrow \texttt{ct}$. On input a key $\kappa \in {\mathcal{K}}$ and a message $\texttt{msg}$, it outputs a ciphertext $\texttt{ct}$.
- ${\textsf{Dec}}(\kappa , \texttt{ct})\rightarrow \mathtt{msg'}$. On input a key $\kappa \in {\mathcal{K}}$ and a ciphertext $\texttt{ct}$, it outputs a recovered message $\mathtt{msg'}$.

Now we give a concrete construction of ROABE as follows.

- ${\textsf{Setup}}(\lambda , {n}, {t})$. On input a security parameter $\lambda$, the number of cloud servers *n* and a threshold t, the algorithm chooses a bilinear map $e:{\mathbb{G}}\times {\mathbb{G}}\rightarrow {\mathbb{G}}_{\text{T}}$, where ${\mathbb{G}}$ and ${\mathbb{G}}_{\text{T}}$ are cyclic groups of $\lambda$-bit prime order *p* with a generator $g\in {\mathbb{G}}$. Then it chooses $g_c, h_c{\mathop {\leftarrow }\limits ^{\$}}{\mathbb{G}}$, $\mu ,\nu {\mathop {\leftarrow }\limits ^{\$}}{\mathbb{Z}}_{p}$ and computes $h=g^\nu$. Subsequently, it chooses two hash functions $\mathsf{H_1}:\{0,1\}^{*}\rightarrow {\mathbb{G}}$, $\mathsf{H_2}:\{0,1\}^{*}\rightarrow {\mathbb{Z}}_{p}$, a key derivation function ${\textsf{KDF}}(\upsilon , L)\rightarrow \{0,1\}^{L}$ where $\upsilon$ is a value that sampled from a source of keying material and $\ell$ is the output length of ${\textsf{KDF}}$, and an SKE with the key space ${\mathcal{K}}$ where $|{\mathcal{K}}|=2^{L}$. In particular, the source of keying material in ROABE is ${\mathbb{G}}_{\text{T}}$. It sets ${\texttt{pk}}=({\mathbb{G}},e, g,h,g_c,h_c,e(g,g)^\mu , \mathsf{H_1}, \mathsf{H_2}, {\textsf{KDF}}, L)$ as a public key and $\texttt{msk}=(\texttt{pk}, \mu , \nu )$ as a master secret key. To generate cloud-side keys, it computes $k_{cs}=g^{\gamma }$ where $\gamma {\mathop {\leftarrow }\limits ^{\$}}{\mathbb{Z}}_{p}$ and randomly defines a polynomial *P*(*x*) over ${\mathbb{Z}}_{p}$ with degree $t-1$ where $P(0)=\gamma$. $\forall i,j\in [n], j\ne i$, it randomly chooses a point $(x_i,y_i)$ over *P*(*x*) where $y_i=P(x_i)$ and $b_{j,i},c_{j,i} {\mathop {\leftarrow }\limits ^{\$}}{\mathbb{Z}}_{p}$, and computes $z_{j,i}=b_{j,i}\cdot y_{i}+c_{j,i}$. It sets ${\texttt{cpk}} = ({\texttt{pk}}, k_{cs}, \{x_i\}_{i\in [n]})$ as a cloud-side public key, and ${\texttt{csk}}_i = ({\texttt{pk}}, dk_i=(y_i,\{z_{j,i}\}_{j\ne i, j\in [n]}), vk_i=\{(b_{i,j},c_{i,j})\}_{j\ne i, j\in [n]})$ as a cloud-side secret key of the *i*th cloud, where $dk_i$ is used to help with decryption and $vk_i$ is used for verification. Finally, it initializes an empty delegated key list ${\mathcal{L}}$ and outputs $({\texttt{pk}}, {\texttt{msk}}, {\texttt{cpk}}, \{{\texttt{csk}}_{i}\}_{i\in [n]}, {\mathcal{L}})$.
- ${\textsf{UKeyGen}}(\texttt{pk},u)$. On input a public key $\texttt{pk}$ and an identity u, the algorithm picks $a_u {\mathop {\leftarrow }\limits ^{\$}}{\mathbb{Z}}_{p}$ and outputs a user’s public/secret key pair $({\texttt{upk}}=g^{a_u}, {\texttt{usk}}=a_u)$.
- ${\textsf{DKeyGen}}(\texttt{msk}, \texttt{cpk}, \texttt{upk}, {\mathcal{R}}, {{\mathcal{L}}})$. On input a master secret key $\texttt{msk}$, a cloud-side public key $\texttt{cpk}$, a user public key ${\texttt{upk}}=g^{a_u}$, a *k*-sized attribute set ${\mathcal{R}}=\{R_1,R_2,...,R_k\}$ and a delegated key list ${\mathcal{L}}$, the algorithm picks $r,r',r_i{\mathop {\leftarrow }\limits ^{\$}}{\mathbb{Z}}_{p}, \forall i\in [k]$ and computes
  $$\begin{gathered} K = \left( {k_{{cs}}^{r} \left( {g^{{a_{u} }} } \right)^{\mu } g^{{r^{\prime } }} } \right)^{{\frac{1}{\nu }}} = g^{{\frac{{r\gamma + \mu a_{u} + r^{\prime } }}{\nu }}} ,K^{\prime } = g^{r} \hfill \\ K_{{i,1}} = g^{{r^{\prime } }} H_{1} (R_{i} )^{{r_{i} }} ,\;K_{{i,2}} = g^{{r_{i} }} ,\;\forall i \in [k],R_{i} \in \mathcal{R} \hfill \\ \end{gathered}$$
  Note that $k_{cs}=g^{\gamma }$ is contained in $\texttt{cpk}$. Above all, it sets a delegated key ${\texttt{dk}}=({\mathcal{R}}, K, K', \{K_{i,1}, K_{i,2}\}_{i\in [k]})$ and adds the entry $({u}, \texttt{dk})$ to ${\mathcal{L}}$ to get an updated list ${\mathcal{L}}'$. Finally, it outputs $\texttt{dk}$ and ${\mathcal{L}}'$.
- ${\textsf{Encrypt}}(\texttt{pk},\mathcal{T}, \texttt{m})$. On input a $\texttt{pk}$, an access tree $\mathcal{T}$ and a message $\texttt{m}$, for each node $\omega$ of $\mathcal{T}$, the algorithm chooses a polynomial $\theta _\omega$ with degree $d_\omega =th_\omega -1$ where $th_\omega$ is the threshold of $\omega$ as follows: it sets $\theta _\omega (0)=\theta _{pt(\omega )}(idx(\omega ))$ and randomly chooses other $d_\omega$ points to completely define $\theta _\omega$. For the root node $\omega _{rt}$, it picks $s,\xi {\mathop {\leftarrow }\limits ^{\$}}{\mathbb{Z}}_{p}$ and sets $\theta _{\omega _{rt}}(0)=s$. Let ${\mathcal{J}}$ be the set of leaf nodes, it computes $C=h^s=g^{\nu s}$, $C'=g^s$, $Y=e(g, g)^{\mu s}$ and
  $$\begin{aligned}&X={\textsc {SKE}}.{\textsf{Enc}}({\textsf{KDF}}(Y,L), m||\xi ), ~~~{\hat{C}}=g_c^{\mathsf{H_2}(\texttt{m})}h_c^{\xi } \\&C_{j,1}=g^{\theta _j(0)}, ~~~C_{j,2}=\mathsf{H_1}(A(j))^{\theta _j(0)}, ~~\forall j\in {\mathcal{J}}. \end{aligned}$$
  Finally, the algorithm outputs a ciphertext ${\texttt{ct}}=(\mathcal{T}, X, C, C',{\hat{C}}, \{C_{j,1},C_{j,2}\}_{j\in {\mathcal{J}}})$.
- ${\textsf{DSGen}}({\texttt{csk}}_{j}, {\texttt{dk}}, i, \texttt{ct})$. On input a cloud-side secret key ${\texttt{csk}}_{j}$ corresponding to a serial number *j*, a delegated key $\texttt{dk}$, a cloud’s serial number *i* and a ciphertext $\texttt{ct}$, the algorithm outputs a decryption share ${\texttt{ds}}_{j,i}= e(C',K')^{y_{j}}=e(g, g)^{s r y_{j}}$ and a corresponding proof ${\pi }_{j,i}=e(C',K')^{z_{i,j}}=e(g,g)^{s r z_{i,j}}$ if the attribute set ${\mathcal{R}}$ in $\texttt{dk}$ satisfies the access tree in $\texttt{ct}$. Otherwise, it outputs $\bot$.
- ${\textsf{DSVerify}}({\texttt{csk}}_{i}, {\texttt{dk}}, {\texttt{ds}}_{j,i}, {\pi }_{j,i}, \texttt{ct})$. On input a ${\texttt{csk}}_{i}$, a $\texttt{dk}$, a decryption share ${\texttt{ds}}_{j,i}=e(g, g)^{s r y_{j}}$, a proof ${\pi }_{j,i}$ and a $\texttt{ct}$, the algorithm gets the terms $(b_{i,j},c_{i,j})$ which is indexed by *j* in ${\texttt{csk}}_{i}$. Then it checks ${\pi }_{j,i}{\mathop {=}\limits ^{?}}(e(g, g)^{s r y_{j}})^{b_{i,j}}\cdot e(C',K')^{c_{i,j}}$. If the equation holds, it outputs $b=1$; otherwise, $b=0$.
- ${\textsf{DSCombine}}({\texttt{cpk}}, \{{\texttt{ds}}_{j,i}\}_{j\in {\Phi }_{t}})$. On input a cloud-side public key $\texttt{cpk}$ and t decryption shares $\{{\texttt{ds}}_{j,i}\}_{j\in {\Phi }_{t}}$ where ${\texttt{ds}}_{j,i}=e(g, g)^{s r y_{j}}$, ${\Phi }_{t}\subseteq [n]$ and $|{\Phi }_{t}|=t$, the algorithm sets ${\mathcal{X}} = \{x_j|j\in {\Phi }_{t}\}$ where $x_j$ is in $\texttt{cpk}$ and computes the Lagrange coefficient $\eta _j = \Delta _{j,{{\mathcal{X}}}}(0)=\prod \limits _{x\in {\mathcal{X}},x \ne j}\frac{0-x}{j-x}, \forall j\in {\Phi }_{t}$. Finally, it outputs a combined secret value
  $$\begin{aligned} {\texttt{csv}}=\prod \limits _{j\in {\Phi }_{t}}(e(g, g)^{s r y_{j}})^{\eta _j}=e(g,g)^{s r P(0)}=e(g,g)^{s r \gamma }. \end{aligned}$$
- ${\textsf{CSDecrypt}}(\texttt{pk}, \texttt{dk}, \texttt{ct}, \texttt{csv})$. On input a $\texttt{pk}$, a $\texttt{dk}$, a $\texttt{ct}$ and a combined secret value ${\texttt{csv}}=e(g,g)^{s r \gamma }$, the algorithm outputs $\bot$ if $Q(\mathcal{T},{\mathcal{R}})\ne 1$. Note that $Q(\mathcal{T},{\mathcal{R}})\ne 1$ means ${\mathcal{R}}$ does not satisfy the access tree $\mathcal{T}$. Otherwise, for each leaf node *j*, if there exists an index *i* s.t. $A(j)=R_{i}\in {\mathcal{R}}$, it sets a node function $D_j = \frac{e(K_{i,1},C_{j,1})}{e(C_{j,2},K_{i,2})}=e(g,g)^{r' \theta _j(0)}$; otherwise, it sets $D_j=\bot$. Then for each non-leaf node $\omega$, it recursively sets the node function $D_\omega$ as follows: let ${\mathcal{J}}_{\omega }$ be a child nodes set of $\omega$ with size $th_\omega$, it tries to find a set ${\mathcal{J}}_{\omega }$ s.t. $D_{\omega _c}\ne \bot$ for any child node $\omega _c\in {\mathcal{J}}_{\omega }$. If no such ${\mathcal{J}}_{\omega }$, $D_\omega =\bot$. Otherwise, let ${\mathcal{I}}_{\omega }=\{idx(\omega _c) | \omega _c \in {\mathcal{J}}_{\omega }\}$, it uses polynomial interpolation to compute
  $$\begin{aligned} D_\omega&= \prod _{\omega _c\in {\mathcal{J}}_{\omega }}(D_{\omega _c})^{\eta _{\omega _c}}, ~\hbox {where} ~ \eta _{\omega _c} = \Delta _{\delta ,{\mathcal{I}}_{\omega }}(0), \delta =idx(\omega _c) \\&=\prod _{\omega _c\in {\mathcal{J}}_{\omega }}(e(g,g)^{r' \theta _{\omega _c}(0)})^{\eta _{\omega _c}} \\&=\prod _{\omega _c\in {\mathcal{J}}_{\omega }}(e(g,g)^{r' \theta _{\omega }(\delta )})^{\eta _{\omega _c}}=e(g,g)^{r' \theta _{\omega }(0)}. \end{aligned}$$
  Then it has $D_{\omega _{rt}}=e(g,g)^{r' \theta _{\omega _{rt}}(0)}=e(g,g)^{r's}$ for the root node and computes $\overline{C}=\frac{e(K,C)}{e(g,g)^{s r \gamma }\cdot D_{\omega _{rt}}}=e(g,g)^{\mu s\cdot a_u}$. Finally, it outputs a partially decrypted ciphertext ${\texttt{dct}}=(X, \overline{C}, {\hat{C}})$. Note that *X* and ${\hat{C}}$ are the terms in $\texttt{ct}$.
- ${\textsf{UDecrypt}}({\texttt{pk}, \texttt{dct}}, \texttt{usk})$. On input a $\texttt{pk}$, a partially decrypted ciphertext $\texttt{dct}$ and a user secret key ${\texttt{usk}}=a_u$, the algorithm computes $Y'={(\overline{C})}^{1/a_u}=(e(g,g)^{\mu s\cdot a_u})^{1/a_u}=e(g,g)^{\mu s}$ and $\mathtt{m'}||\xi '={\textsc {SKE}}.{\textsf{Dec}}({\textsf{KDF}}(Y', L), X)$. It outputs $\mathtt{m'}$ if ${\hat{C}}=g_c^{\mathsf{H_2}(\mathtt{m'})}h_c^{\xi '}$. Otherwise, it outputs $\bot$.
- $\textsf{URevoke}({u}, {{\mathcal{L}}})$. On input an identity u and a delegated key list ${\mathcal{L}}$, the algorithm deletes the entry $({u}, \texttt{dk})$ from ${\mathcal{L}}$ to get an updated list ${\mathcal{L}}'$.

### Security analysis

In this section, we give four theorems with respect to the security definitions and model the hash function $\mathsf{H_1}$ as a random oracle. The security proofs are postponed to appendix.

#### Theorem 1

Our ROABE scheme achieves data privacy against both users (type-1 adversary) and clouds (type-2 adversary) in the generic group model, assuming ${\textsf{KDF}}$ is secure.

#### Theorem 2

Our ROABE scheme achieves reliable user revocation against type-3 adversary in the generic group model, assuming ${\textsf{KDF}}$ is secure.

#### Theorem 3

Our ROABE scheme achieves verifiability of decryption share against type-4 adversary.

#### Theorem 4

Our ROABE scheme achieves verifiability of intermediate ciphertext against type-5 adversary, assuming the Discrete Logarithm (DL) problem is hard in the prime order bilinear group system, ${\textsf{KDF}}$ is secure and the hash function $\mathsf{H_2}$ is collision-resistant.

## Implementation of rainbow

In this section, we present how to build Rainbow with ROABE and other cryptographic and industrial tools, and the deployment in real world. In particular, ROABE, Public Key Infrastructures (PKI), Message Queue (MQ), ownCloud and digital signature are main components in Rainbow. ROABE brings core security properties, which have been defined in the design goals. PKI generates certificates to authenticate system users and the servers and MQ is adopted to transmit the confirmation messages (refer to next subsection). The software ownCloud implies the basic functionalities of cloud storage hosting, e.g., data upload, download, and sharing. To ensure the confirmation message unforgeable, the signature is applied.

Three mainstream clouds are chosen, namely AWS, GCP and Azure, for building multiple cloud servers. The users in Rainbow, including PO and PU, are equipped with browsers and smart phones and client certificates are settled ahead in browsers and the Android application to build the secure channel.

### Detailed construction

**Fig. 3:**
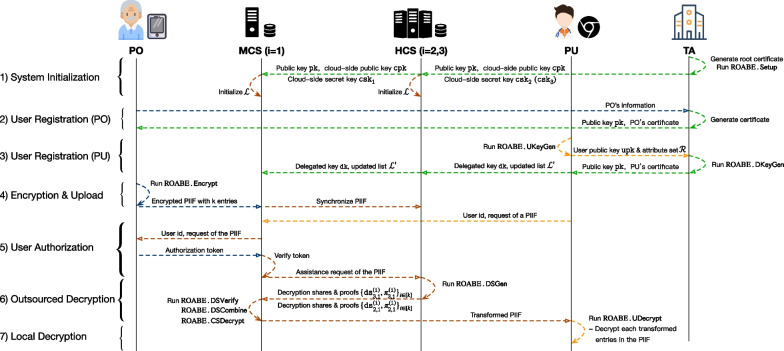
Main interactions in Rainbow

Now we present the details of Rainbow following the workflow. Fig. 3 depicts the interactions of each entity of Rainbow using ROABE where ($n=3$, $t=2$).

1. *System Iiitialization.* TA chooses the threshold value t and labels each cloud server with a unique serial number, e.g., in Fig. 3, the serial number of MCS is 1 and the serial numbers of other two HCSs are 2 and 3, respectively. Then TA initializes cryptographic modules, e.g., using AES-GCM to initialize SKE. For ROABE, a global attribute universe $\mathcal{U}$ that contains all available attributes is set. Taking $\lambda$ as input, ROABE.${\textsf{Setup}}$ is called to get ($\texttt{pk}$, $\texttt{msk}$, $\texttt{cpk}$, $\{{\texttt{csk}}_{i}\}_{i\in [n]}$, ${\mathcal{L}}$). TA also maintains a Public Key Infrastructures (PKI) to issue certificates for users. It generates a root certificate in this phase. Finally, ${\texttt{csk}}_{i}$ is securely transmitted to the *i*th cloud server along with ($\texttt{pk}$, $\texttt{cpk}$). The empty delegated key list ${\mathcal{L}}$ is initialized by each cloud server.
2. *User registration (PO).* A PO generates a signing key and a verification key for signature and sends his registration information, such as identity and contact details, along with the verification key to TA. TA issues the request and generates a certificate with the verification key for the PO. The certificate will be transmitted to the PO.
3. *User registration (PU).* A PU runs ROABE.${\textsf{UKeyGen}}$ with his identity u to obtain ($\texttt{upk}$,$\texttt{usk}$). Then he also generates a signing key and a verification key for signature and sends a registration request which contains $\texttt{upk}$, an attribute set ${\mathcal{R}}$ and the verification key to TA. TA also generates a certificate for him and runs ROABE.${\textsf{DKeyGen}}$ to produce a delegated key $\texttt{dk}$. The new entry (u, $\texttt{dk}$) is added into ${\mathcal{L}}$ by each cloud server. Note that the certificate of PO/PU is used for confirmation (see Phase 5)
4. *Encryption and upload.* For each entry in a PO’s PIIF, he chooses an access policy and runs ROABE.${\textsf{Encrypt}}$ to get a ciphertext $\texttt{ct}$. Note that each entry in a PIIF is in “key-value” style like JSON, e.g., {key: Name, value: Alice}, and only value (e.g., Alice) is encrypted. Combining all encrypted entries, he forms an encrypted PIIF and uploads it to a cloud server. The encrypted PIIF are synchronized to other servers to make a backup.
5. *Owner confirmation.* In Rainbow, before a PU obtaining an encrypted PIIF, he should get the confirmation from the PO. In particular, the PU signs his request with the signing key and sends the request along with his certificate and the signature to a cloud server (MCS), where the request contains his identity, purpose, requested PIIF, etc. The MCS checks the validity of the signature and rejects if it is invalid or the PU is not in ${\mathcal{L}}$. Otherwise, it pushes the request to the PO. If the PO allows the PU to access this PIIF, he generates a confirmation (or rejection) token and signs it. The token and the signature are returned to the MCS.
6. *Outsourced Decryption.* The MCS checks the validity of token with PO’s certificate and generates an assistance request of the target PIIF if the token is valid and sends the request to other t HCSs. When an HCS, whose serial number is *j*, receives the request from the MCS, it refuses to help if the PU is not in ${\mathcal{L}}$. Otherwise, suppose there are *k* encrypted entries in the requested PIIF, for the *i*th entry, it runs ROABE.${\textsf{DSGen}}$ to generate $({\texttt{ds}}_{j,1}^{(i)}, {\pi }_{j,1}^{(i)})$. Recall that the serial numer of MCS is 1 in Fig. 3. The HCS returns the set $\{({\texttt{ds}}_{j,1}^{(i)}, {\pi }_{j,1}^{(i)})\}_{i\in [k]}$ to the MCS. For each tuple in the set, the MCS runs ROABE.${\textsf{DSVerify}}$ to check the correctness of ${\texttt{ds}}_{j,1}^{(i)}$. If it is invalid, the MCS would choose another HCS with a serial number $j^*$ $(j^*\ne j)$ that has not been requested in this session to obtain a new tuple. The misbehavior of this HCS would be recorded. Once the MCS gets t valid decryption shares for an encrypted entry, it runs ROABE.${\textsf{DSCombine}}$ and ROABE.${\textsf{CSDecrypt}}$ to get a partially decrypted ciphertext $\texttt{dct}$. Finally, combining all transformed ciphertexts, it forms a transformed PIIF and returns it to the PU.
7. *Local decryption.* When the transformed PIIF received, for each partially decrypted entry, the PU runs ROABE.${\textsf{UDecrypt}}$ to obtain a plaintext or $\bot$. If $\bot$ is output, the MCS is caught as a misbehaved server since the partially decrypted ciphertext $\texttt{dct}$ is not well-formed. Otherwise, the recovered entries in the PIIF can be reconstructed as a decrypted PIIF. We stress that the PU maybe cannot decrypt all entries in the PIIF due to the access policy of each entry.
8. *User revocation.* Once a PU u is suggested to be revoked, all cloud servers should run ROABE.${\textsf{URevoke}}$ to remove the entry (u,$\texttt{dk}$) from ${\mathcal{L}}$. Then the cloud servers cannot provide outsourced decryption for u anymore. Besides, TA would revoke his certificate as well.

### Adapting rainbow with ownCloud

**Fig. 4:**
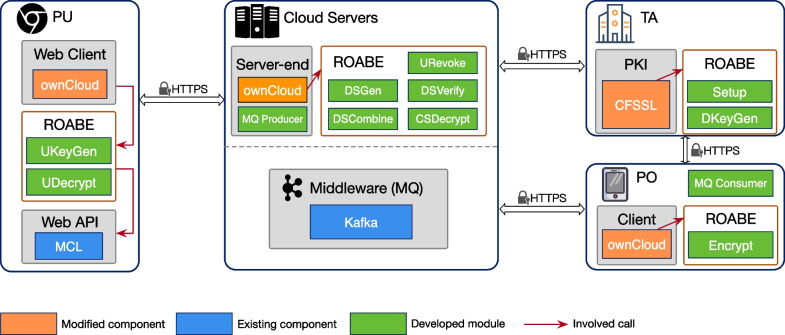
System architecture

In this section, we introduce how to adapt Rainbow with ownCloud. Besides, some practical middlewares and tools are used to initialize Rainbow in real world. The architecture is shown in Fig. 4.

We chose the BN curve (Barreto and Naehrig , 2005) and implemented each algorithm in ROABE using MCL library (Mitsunari , 2019). We also used AES-GCM from OpenSSL to instantiate SKE. All the algorithms were compiled to a dynamic library (.so). For the ownCloud server, we used PHP-CPP (http://www.php-cpp.com/) to transform our dynamic library to a PHP extension. For the Android client, we adopted Java Native Interface (JNI) (https://docs.oracle.com/javase/8/docs/technotes/guides/jni/) and cross-compilation technique to repackage APIs to fit Android OS. And for the web application, we used JavaScript to implement algorithms by adopting WebAssembly (W3C Community Group , 2017) and MCL-WASM (Mitsunari , 2019). Besides, we used JSON, which is one of the most popular data-interchange formats, to form the PIIF. More details are given here.

*TA.* We used CFSSL (CloudFlare , 2014) which is cloudflare’s PKI and TLS toolkit to build the PKI in TA. To deploy the algorithms of ROABE to TA, including ${\textsf{Setup}}$ and ${\textsf{DKeyGen}}$, we modified CFSSL using CGO, which enables the creation of Go packages that call C code.

*Cloud servers.* As we discussed above, the algorithms of ROABE are embedded into the ownCloud server, including ${\textsf{DSGen}}$, ${\textsf{DSVerify}}$, ${\textsf{DSCombine}}$, ${\textsf{CSDecrypt}}$ and ${\textsf{URevoke}}$. To guarantee the confirmation request can be pushed to the PO in time, we adopted the middleware message queue (MQ), namely Kafka (Apache 2011), and deployed it on cloud servers. Therefore, we additionally built an MQ producer module to ownCloud server to transmit the requests from users.

*User side.* Android client and web application are modified to adapt with ROABE.

- *Android client.* It is considered as a PO. The algorithm ${\textsf{Encrypt}}$ was implemented and exposed to ownCloud via JNI. We additionally built an MQ consumer module to fetch the transmitted requests from Kafka.
- *Web application.* The algorithms of ROABE, namely ${\textsf{UKeyGen}}$ and ${\textsf{UDecrypt}}$, were implemented by using JavaScript and WebAssembly-based API from MCL.

### System deployment

We now present how to deploy Rainbow in real world.

*Basic clouds setting.* We adopted AWS, GCP and Azure as the cloud service provider. To use their services, they mandate that we should create cloud accounts and follow their access control rules, e.g., ABAC and RBAC (https://docs.aws.amazon.com/IAM/latest/UserGuide/introduction_attribute-based-access-control.html, https://cloud.google.com/iam/docs/overview, https://docs.microsoft.com/en-us/azure/role-based-access-control/overview). A trivial idea is binding a user to a corresponding account on each cloud, however, it is impractical since we have to build an authentication module to link the access rights of system users and cloud accounts. Instead, we created only an account on each cloud that have definite access rights and binded this account to the ownCloud server. Then the user’s access rights are fully controlled by Rainbow, which are independent with the cloud service providers.

*Network configuration.* The channels between all entities are protected by TLS protocol with public key certificates thus bi-directional authentication is promised. We adopt VPN as the internal channel for the communication between each cloud server. All clients access to the clouds through the public network.

*Instance deployment.* We deployed our modified ownCloud server on Amazon EC2, Google Compute Engine and Azure Virtual Machines. Specifically, we appreciate to adopt Trusted Execution Environment (TEE) (http://www.omtp.org/OMTP_Advanced_Trusted_Environment_OMTP_TR1_v1_1.pdf) to protect the computation on TA, however, it is out of our concern in this work. We used Amazon S3, Google Drive and Azure File Storage as external storage services of ownCloud. We installed the modified Android client on smart phones to perform as system users.

## System evaluation

### Theoretical comparison

For Rainbow, the majority of computation cost and security functionalities come from ROABE. In Table 2, we compare ROABE to other known schemes in three folds, including functionality, security model and basic computation cost.

**Table 2:** Comparisons of Some CP-ABE Schemes

Scheme	User revocation		Security		Full verifiability	User decryption computation
	Immediateness	Reliability	Trust on cloud server	Model of attack		
Attrapadung and Imai (2009)	$\checkmark$	$\times$	–	Selective	$\times$	$3|I|E+4|I|P$
Cui et al. (2016)	$\times$	$\times$	Untrust	Selective	$\times$	$1E'$
Qin et al. (2017)	$\times$	$\times$	Untrust	Selective	$\times$	2*P*
Ma et al. (2015)	–	-	Covert	Selective	$\Delta$	$1E'$
Ma et al. (2019)	$\checkmark$	$\times$	Semi-honest	Selective	$\times$	$1E'$
Yang et al. (2015)	$\checkmark$	$\times$	Semi-honest	Adaptive	$\times$	$1E'$
Our ROABE	$\checkmark$	$\checkmark$	Covert	Adaptive	$\checkmark$	$1E'$

“–” denotes “not applicable”. “$\times$” denotes “not support”, “$\Delta$” denotes “partially support” and “$\checkmark$” denote “fully support”. “|*I*|” are the cardinality of the satisfied attribute set. *E*, $E'$, *P* are the numbers of modular exponentiations in ${\mathbb{G}}$ and ${\mathbb{G}}_{\text{T}}$, and paring, respectively

Attrapadung and Imai (2009) put forward a direct user revocation that revokes a user directly by incorporating a revocation list into encryption. However, it is heavy to revoke a user from the system since all ciphertexts need to be updated. ROABE achieves efficient and immediate user revocation via server-aided approach (Yang et al. 2015; Ma et al. 2019). Although Cui et al. (2016) and Qin et al. (2017) gave server-aided solutions, they need to update all other users’ delegated keys when a user revoked, which is impractical. The user revocation mechanism proposed by Ma et al. (2019) does not fully support the reliability if multiple servers are deployed in practice. Because there is only one cloud-side secret key holding by all servers. The mechanism in Ma et al. (2019) can resist the leakage of cloud-side secret key by updating ciphertexts, nevertheless, it costs too much. Besides, the works (Cui et al. 2016; Qin et al. 2017) can also achieve key-exposure, but updating all delegated keys is demanded when leakage occurs. Our scheme can resist key-exposure since unless the adversary obtains more than t cloud-side secret keys, it is unable to break the revocation. Above all, the schemes (Attrapadung and Imai 2009; Cui et al. 2016; Qin et al. 2017; Ma et al. 2019; Yang et al. 2015) and our ROABE all support user revocation, only ROABE achieves immediateness and reliability simultaneously, nevertheless.

Our ROABE achieves full verifiability to check the correctness of outsourced decryption and locate a misbehaved server when a wrong decryption result returned, while the verification mechanism proposed by Ma et al. (2015) only supports the former. The works (Lai et al. 2013; Mao et al. 2015; Lin et al. 2016) have the same limitation. Since none of them can accurately locate the misbehaved server over multi-cloud, we conclude that they “partially support” the full verifiability. Besides, ROABE is efficient on user side since only one exponentiation operation is required for local decryption. The above theoretical comparison shows that our scheme is practical and secure.

### Feature discussion

In this subsection, we further discuss the features of Rainbow.

*Reliable immediate user revocation.* The user revocation in Rainbow is immediate since it is only required to remove a PU’s delegated key from the list ${\mathcal{L}}$ on each server. The reliability lies in two folds. One is key-exposure resistance. When no more than $t-1$ servers compromised, the revocation mechanism still works, referring to the security property of ROABE. The other is high availability. Even several servers (less than $n-t$) collapse, Rainbow can still provide retrieval service as well as user revocation. In particular, in the outsourced decryption phase, a PU can adaptively choose other servers as MCS when the requested MCS collapses, and the MCS can choose other alive servers as its HCS until *t* valid decryption shares are obtained when any HCS collapses.

*Accurate Judgement for misbehaved outsourced decryption.* In Rainbow, a misbehaved server cannot exculpate itself for a wrong outsourced decryption result. In particular, suppose an HCS produces an incorrect decryption share, the MCS can check its correctness and disclose its misbehavior according to the verifiability of decryption share of ROABE. If the MCS shields the HCS, since ROABE implies the verifiability of partially decrypted ciphertext and a wrong decryption share would cause an incorrect $\texttt{dct}$, the PU could blame the MCS for its misbehavior. Although the misbehaved HCS conceals himself in this case, the MCS is located and punished, and the wrong result is eventually figured out and never be used. In fact, according to this property, it is worthless for the MCS to shield a misbehaved HCS. Hence, no misbehaved server can exculpate itself.

*More security properties.* Regarding to the involved components, Rainbow additionally brings the following security properties.

1. *Secure communication.* Since PKI generates certificates for system users, the communication channel between each entity can be easily secured by implementing TLS. Besides, the message queue, i.e., Kafka, also implies secure communication by setting TLS/SSL configuration.
2. *Undeniable confirmation token.* In the owner confirmation phase, the PU would generate a confirmation token and send it to the MCS along with a corresponding signature. It prevents any PU from denying the retrieval request of PIIF, which gives a promising solution to trace unexpected PIIF leakage in real world.
3. *Field-level access control.* In Rainbow, each entry in PIIF can be encrypted independently with arbitrary access policy. It implies field-level access control.

### Experimental results

**Fig. 5:**
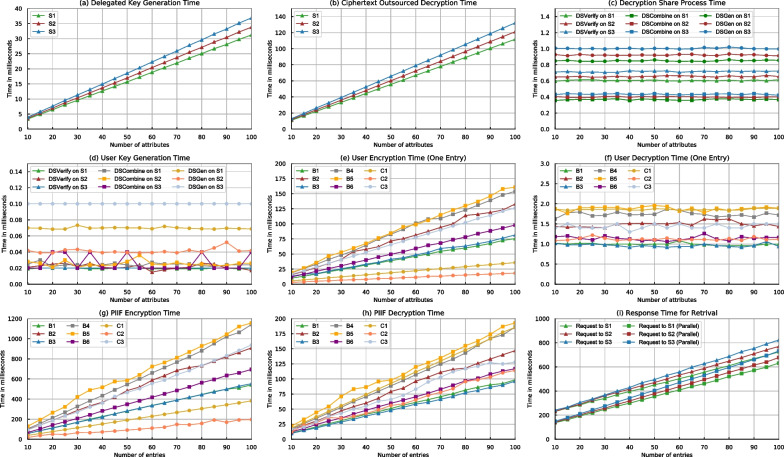
Experimental performance

To evaluate the performance of Rainbow, we hired several cloud service providers, namely Amazon, Google and Microsoft, and used various user devices, including laptop, desktop and mobile phone, as our experimental subjects (see Table 3). We used AES-GCM-128 to instantiate SKE and PBKDF2-HMAC-SHA256 to realize ${\textsf{KDF}}$ which outputs 128bit derived key. The signature is implemented by the Boneh-Lynn-Shacham scheme (Boneh et al. , 2001). All experimental results are shown in Fig. 5 and all times are presented in milliseconds (ms). In particular, the experiment contains two parts: the raw performance of ROABE (Fig. 5a–f) and the performance of Rainbow based on ownCloud (Fig. 5g–i).

**Table 3:** Experiment setup

	Label	CPU	OS	Type
Client	C1	Intel Core(TM) i7-3770 @3.40GHz	Ubuntu 16.04	Desktop
	C2	Intel Core i7-9750H @2.60GHz	MacOS Catalina 10.15	Laptop
	C3	HUAWEI Kirin 990E @2.86GHz	HarmonyOS 2.0.0	Mobile
Server	S1	Intel Xeon Platinum 8272CL @2.60GHz	Centos 7	Azure
	S2	Intel Xeon E5-2676 v3 @2.40GHz	Amazon Linux 2	AWS
	S3	Intel Xeon E5-2650 v4 @2.20GHz	Centos 7	GCP

Device	Label	Browser
Macbook Pro Intel Core i7-9750H @2.60GHz	B1	Safari 15609.4.1
	B2	Chrome 106.0.5249.119
	B3	Firefox 102.0.1
Dell Laptop Intel Core i7-8550U @1.80GHz	B4	Microsoft Edge 107.0.1418.26
	B5	Chrome 107.0.5304.88
	B6	Firefox 106.0.3

To evaluate the performance of algorithms in ROABE, we set access policies in the form of $(R_1\wedge R_2 \wedge \cdots \wedge R_\ell )$ to simulate the worst case. We set 20 distinct access policies with $\ell$ increasing from 5 to 100, repeat each instance 50 times and take the average value. Figure 5a shows that the key generation costs 1.8–36.8 ms which performs well on different servers with different operating systems. As shown in Fig. 5b, the running time of ${\textsf{CSDecrypt}}$ is about 5.4–132.1 ms on three cloud servers (S1–S3). Figure 5c shows that ${\textsf{DSGen}}$, ${\textsf{DSVerify}}$ and ${\textsf{DSCombine}}$ cost about 0.8–1.0 ms, 0.6–0.7 ms and 0.3–0.4 ms, respectively. Comparing Fig. 5b, c, the process of the decryption share costs much less than ${\textsf{CSDecrypt}}$. It indicates that we can run these algorithms in parallel setting to further optimize the performance on servers. We discuss about the optimization in Rainbow later. Figure 5d shows that the running time of ${\textsf{UKeyGen}}$ is about 0.02–0.04 ms in browsers (B1–B6) and 0.04–0.1 ms on clients (C1–C3). Figure 5e demonstrates that ${\textsf{Encrypt}}$ costs about 6.5–161.8 ms in browsers and 2.0–127.1 ms on clients. Figure 5f indicates that the running time of ${\textsf{UDecrypt}}$ is independent of the number of attributes. It costs about 1.0–1.8 ms in browsers and 1.1–1.5 ms on clients.

To evaluate the performance of Rainbow, we produced multiple PIIFs which are formed in JSON and mainly tested user-side performance, including encryption and local decryption, and response latency of retrieval. We increased the number of contained entries from 5 to 100 and set the length of each entry to be 20 bytes. The policy of each entry was set in the form of $(R_1\wedge R_2 \wedge R_3 \wedge R_4 \wedge R_5)$, where the number of attributes is fixed to be 5. Figure 5g, h indicate that the encryption costs about 28.6–1158.0ms and the decryption costs about 5.2–193.6ms in browsers. Even 100 entries are contained, the encryption costs less than 1.2 s and the decryption costs within 200ms. The network latency between each cloud server is about 15ms on average and the public bandwidth is 1Mbps. Figure 5i shows the response latency of the cloud server is affordable when a PU sends his request where $(n=5, t=3)$. In particular, it contains confirmation and outsourced decryption. To optimize the performance on server, in outsourced decryption phase, Rainbow generates assistance requests and runs ${\textsf{CSDecrypt}}$ simultaneously with different processes. When decryption shares are returned from other servers, as shown in Fig. 5b, c, ${\textsf{CSDecrypt}}$ possibly has not finished. And we observed that the output of ${\textsf{DSCombine}}$, namely $\texttt{csv}$, is used at the last step of ${\textsf{CSDecrypt}}$. Therefore, Rainbow can create another process to deal with ${\textsf{DSVerify}}$ and ${\textsf{DSCombine}}$ and pass $\texttt{csv}$ to the main process which are still running ${\textsf{CSDecrypt}}$ via internal process communication (IPC). The optimized results are shown in Fig. 5i. All experimental results indicate that Rainbow is practical.

## Conclusion

In this paper, we propose Rainbow, a secure and practical PII retrieval scheme. As a step towards our construction and a by-product, we design and implement a useful tool called ROABE with data privacy, flexible and fine-grained access control, reliable immediate user revocation and verification for multiple servers. Then we present a formal security model and give theoretical security analysis of ROABE. With ROABE, ownCloud, a popular cloud storage hosting application, and other cloud techniques, we implement Rainbow in real world. To evaluate its performance, we deploy Rainbow on multiple mainstream clouds, namely AWS, GCP and Azure, and different clients and browsers. Combining the security analysis and the experimental evaluation, we conclude that Rainbow achieves great performance with enhanced security guarantees.

## Data Availability

Not applicable.

## Appendix

### Proof of Theorem 1

In this section, we give our proofs of data privacy against users and clouds, respectively, to prove Theorem 1. Let $\Pi$ be the scheme in Yang et al. (2015).

#### Data privacy against users

##### Proof

We define the following games.

- $\texttt{Game}_{\text{0}}^{\text{}}$: It is the original security game $\texttt{Game}_{\text{priv}}^{\text{du}}$.
- $\texttt{Game}_{\text{1}}^{\text{}}$: Almost same as $\texttt{Game}_{\text{0}}^{\text{}}$, except that $Y{\mathop {\leftarrow }\limits ^{\$}}{\mathbb{G}}_{\text{T}}$, $X^*={\textsc {SKE}}.{\textsf{Enc}}({\textsf{KDF}}(Y, L), \texttt{m}_b||\xi )$.
- $$\texttt{Game}_{\text{2}}^{\text{}}$$: Almost same as $$\texttt{Game}_{\text{1}}^{\text{}}$$, except that $$X^*=$$ SKE.$${\textsf{Enc}}$$$$(K, \texttt{m}_b||\xi )$$ where *K* is randomly picked from the key space of SKE.
- $\texttt{Game}_{\text{3}}^{\text{}}$: Almost same as $\texttt{Game}_{\text{2}}^{\text{}}$, except that $X^*=$ SKE.${\textsf{Enc}}$(*K*, *M*) where *M* is randomly picked from the message space of SKE and $|M|=|\texttt{m}_b|+|\xi |$.
- $\texttt{Game}_{\text{4}}^{\text{}}$: Almost same as $\texttt{Game}_{\text{3}}^{\text{}}$, except that ${\hat{C}}^*=g_c^{\mathsf{H_2}(x)}h_c^{\xi }$ where *x* is a randomness.

##### Lemma 1

$\texttt{Game}_{\text{0}}^{\text{}}$ and $\texttt{Game}_{\text{1}}^{\text{}}$ are indistinguishable, if $\Pi$ achieves data privacy against users.

##### Proof

Assume that there exists a PPT adversary $\mathcal{A}$ who can distinguish $\texttt{Game}_{\text{0}}^{\text{}}$ and $\texttt{Game}_{\text{1}}^{\text{}}$ with a non-negligible advantage $\epsilon$, then we can build a PPT simulator $\mathcal{B}$ to break the data privacy against users of $\Pi$ with a non-negligible advantage $\epsilon '$. Let $\mathcal{C}$ be the challenger of $\Pi$.

${{\textsc {Setup.}}}$ $\mathcal{C}$ generates public parameters $params=({\mathbb{G}}, e, g, h=g^{\nu }, e(g,g)^{\mu }, \mathsf{H_1})$ and a cloud’s key pair $(pk_{cs}=g^{\gamma }, sk_{cs}=\gamma )$, and returns them to $\mathcal{B}$. Then $\mathcal{B}$ picks $({t},n)$ where $t\le n$, and chooses $g_c, h_c{\mathop {\leftarrow }\limits ^{\$}}{\mathbb{G}}$, a hash function $\mathsf{H_2}:\{0,1\}^{*}\rightarrow {\mathbb{Z}}_{p}$ and a key derivation function ${\textsf{KDF}}$ with the output length *L*. $\mathcal{B}$ defines a polynomial *P*(*x*) over ${\mathbb{Z}}_{p}$ with degree ${t}-1$ where $P(0)=\gamma$. $\forall i,j\in [n], j\ne i$, it randomly chooses a point $(x_{i},y_{i})$ over *P*(*x*) where $y_{i}=P(x_{i})$ and $b_{j,i},c_{j,i} {\mathop {\leftarrow }\limits ^{\$}}{\mathbb{Z}}_{p}$, and computes $z_{j,i}=b_{j,i}\cdot y_{i}+c_{j,i}$. $\mathcal{B}$ sets $\texttt{pk}=(params, g_c, h_c, \mathsf{H_2}, {\textsf{KDF}}, L)$, $\texttt{cpk} = (\texttt{pk}, k_{cs}, \{x_i\}_{i\in [n]})$ and $\texttt{csk}_{i} = (\texttt{pk}, dk_i=(y_i,\{z_{j,i}\}_{j\ne i, j\in [n]}), vk_i=\{(b_{i,j},c_{i,j})\}_{j\ne i, j\in [n]})$. Finally, $\mathcal{B}$ initializes an empty delegated key list ${\mathcal{L}}$ and a table $\mathcal{W}$, and returns ($\texttt{pk}$, $\texttt{cpk}$, $\{\texttt{csk}_{i}\}_{i\in [n]}$, ${\mathcal{L}}$) to $\mathcal{A}$.

${{\textsc {Phase 1.}}}$ $\mathcal{A}$ is allowed to query following oracles:

- *User-key oracle* $\mathcal{O}_{\textit{UK}}(u)$: $\mathcal{B}$ forwards the query to $\mathcal{C}$. $\mathcal{C}$ returns $(\texttt{upk},\texttt{usk})$ to $\mathcal{B}$. Finally, $\mathcal{B}$ stores them to the table $\mathcal{W}$ and returns $(\texttt{upk}, \texttt{usk})$ to $\mathcal{A}$.
- *Delegated-key oracle* $\mathcal{O}_{\textit{DK}}({\mathcal{R}}, u)$: $\mathcal{B}$ rejects if no such $\texttt{upk}$ exists in $\mathcal{W}$. Otherwise, $\mathcal{B}$ sends the attribute set ${\mathcal{R}}$ to $\mathcal{C}$ to get a $\texttt{dk}=({\mathcal{R}}, K, K', \{K_{i,1}, K_{i,2}\}_{i\in [k]})$ where $k=|\mathcal{R}|$, and returns it to $\mathcal{A}$.

${{\textsc {Challenge.}}}$ $\mathcal{A}$ submits two message $\texttt{m}_{0}, \texttt{m}_{1}$ where $|\texttt{m}_{0}|=|\texttt{m}_{1}|$ and an access tree ${\mathcal{T}}^{*}$, subjecting to a restriction that none of the queried attribute set ${\mathcal{R}}$ in Phase 1 satisfies ${\mathcal{T}}^{*}$. $\mathcal{B}$ randomly picks ${\texttt{m}_{0}^{*}}, {\texttt{m}_{1}^{*}}\in {\mathbb{G}}_{\text{T}}$ and sends ${\texttt{m}_{0}^{*}},{\texttt{m}_{1}^{*}}$ to $\mathcal{C}$ to get a ciphertext $(\mathcal{T}^*, c^*={\texttt{m}_{b}^{*}}\cdot e(g,g)^{\mu s}, C^*=h^s, C^{'*}=g^s, \{C_{j,1}^*=g^{\theta _j(0)}, C_{j,2}^*=\mathsf{H_1}(A(j))^{\theta _j(0)}\}_{j\in {\mathcal{J}}})$, where ${\mathcal{J}}$ is the set of leaf nodes. Then $\mathcal{B}$ flips a random coin $b^*$ and computes $Y=c^*/{\texttt{m}_{b^*}^{*}}=\frac{{\texttt{m}_{b}^{*}}}{{\texttt{m}_{b^*}^{*}}}\cdot e(g,g)^{\mu s}$, ${\hat{C}}^*=g_c^{\mathsf{H_2}(\texttt{m}_{b^*})}h_c^{\xi }$ where $\xi {\mathop {\leftarrow }\limits ^{\$}}{\mathbb{Z}}_{p}$ and $X^*={\textsc {SKE}}.{\textsf{Enc}}({\textsf{KDF}}(Y, L), \texttt{m}_{b^*}||\xi )$. Finally, $\mathcal{B}$ sets ${\texttt{ct}^*}=(\mathcal{T}^*, X^*, C^*, C^{'*}, {\hat{C}}^*, \{C_{j,1}^*, C_{j,2}^*\}_{j\in {\mathcal{J}}})$ and returns it to $\mathcal{A}$.

${{\textsc {Phase 2.}}}$ $\mathcal{A}$ continues to query the oracles with the restriction that any queried ${\mathcal{R}}$ does not satisfy ${\mathcal{T}}^{*}$.

${{\textsc {Guess.}}}$ $\mathcal{A}$ outputs a guess $b'\in \{0,1\}$ to indicate that it plays with the game $\texttt{Game}_{b'}$. If $b'=0$, $\mathcal{B}$ outputs $b^*$ as the guess of *b*. Otherwise, $\mathcal{B}$ outputs $1-b^*$.

Apparently, if $b^*=b$, $\mathcal{B}$ has simulated $\texttt{Game}_{\text{0}}^{\text{}}$ properly since $Y=e(g,g)^{\mu s}$; otherwise, it has simulated $\texttt{Game}_{\text{1}}^{\text{}}$ properly since *Y* is a randomness. Then $\mathcal{B}$ can break the data privacy against users of $\Pi$ with the advantage $\epsilon '=\epsilon$ which is non-negligible. Therefore, $\texttt{Game}_{\text{0}}^{\text{}}$ and $\texttt{Game}_{\text{1}}^{\text{}}$ are indistinguishable. $\square$

Since the security of the KDF implies that ${\textsf{KDF}}$(*Y*, *L*) is indistinguishable from a randomly generated key of SKE, then $\texttt{Game}_{\text{1}}^{\text{}}$ and $\texttt{Game}_{\text{2}}^{\text{}}$ are indistinguishable. Since the Pedersen commitment is computationally hiding, $\texttt{Game}_{\text{3}}^{\text{}}$ and $\texttt{Game}_{\text{4}}^{\text{}}$ are indistinguishable. To prove the indistinguishability of $\texttt{Game}_{\text{2}}^{\text{}}$ and $\texttt{Game}_{\text{3}}^{\text{}}$, we have the following lemma.

##### Lemma 2

If SKE is semantically secure, then $\texttt{Game}_{\text{2}}^{\text{}}$ and $\texttt{Game}_{\text{3}}^{\text{}}$ are computationally indistinguishable.

##### Proof

Suppose there exists a PPT adversary $\mathcal{A}$ who can distinguish $\texttt{Game}_{\text{2}}^{\text{}}$ and $\texttt{Game}_{\text{3}}^{\text{}}$ with a non-negligible advantage, then we can build a PPT simulator $\mathcal{B}$ to break the semantic security of SKE. To avoid repetition, we only discuss the Challenge phase. When $\mathcal{A}$ submits $(\texttt{m}_{0}, \texttt{m}_{1})$ to $\mathcal{B}$, $\mathcal{B}$ flips a coin $b^*$ and sends $({\texttt{m}_{0}^{*}},{\texttt{m}_{1}^{*}})=(\texttt{m}_{b^*}||\xi , M)$ to $\mathcal{C}$ (the challenger of SKE) where $|{\texttt{m}_{0}^{*}}|=|{\texttt{m}_{1}^{*}}|$, $\xi {\mathop {\leftarrow }\limits ^{\$}}{\mathbb{Z}}_{p}$ and *M* is randomly chooses from the message space of SKE. $\mathcal{C}$ flips a coin *b*, randomly picks a symmetric key *K* and returns $X^*={\textsc {SKE}}.{\textsf{Enc}}(K, {\texttt{m}_{b}^{*}})$ to $\mathcal{B}$. $\mathcal{B}$ generates other terms to get the challenge ciphertext $\texttt{ct}^*$ and returns it to $\mathcal{A}$. $\mathcal{B}$ sets $b'$ that is output by $\mathcal{A}$ as its guess.

Obviously, if $b=0$, then $\mathcal{B}$ has simulated $\texttt{Game}_{\text{2}}^{\text{}}$; otherwise, it has simulated $\texttt{Game}_{\text{3}}^{\text{}}$. Then $\mathcal{B}$ can break the semantic security of SKE with a non-negligible advantage. Thus, $\texttt{Game}_{\text{2}}^{\text{}}$ and $\texttt{Game}_{\text{3}}^{\text{}}$ are indistinguishable. $\square$

In $\texttt{Game}_{\text{4}}^{\text{}}$, since the information of $\texttt{m}_{b}$ is lost in the challenge ciphertext, the advantage of $\mathcal{A}$ is exactly 0. Thus, ROABE achieves data privacy against users. $\square$

#### Data privacy against clouds

Similarly, we define the following games.

- $\texttt{Game}_{\text{0}}^{\text{}}$: It is the original security game $\texttt{Game}_{\text{priv}}^{\text{cs}}$.
- $\texttt{Game}_{\text{1}}^{\text{}}$: Almost same as $\texttt{Game}_{\text{0}}^{\text{}}$, except that $Y{\mathop {\leftarrow }\limits ^{\$}}{\mathbb{G}}_{\text{T}}$, $X^*={\textsc {SKE}}.{\textsf{Enc}}({\textsf{KDF}}(Y, L), \texttt{m}_b||\xi )$.
- $$\texttt{Game}_{\text{2}}^{\text{}}$$: Almost same as $$\texttt{Game}_{\text{1}}^{\text{}}$$, except that $$X^*=$$ SKE.$${\textsf{Enc}}$$$$(K, \texttt{m}_b||\xi )$$ where *K* is randomly picked from the key space of SKE.
- $\texttt{Game}_{\text{3}}^{\text{}}$: Almost same as $\texttt{Game}_{\text{2}}^{\text{}}$, except that $X^*=$ SKE.${\textsf{Enc}}$(*K*, *M*) where *M* is randomly picked from the message space of SKE and $|M|=|\texttt{m}_b|+|\xi |$.
- $\texttt{Game}_{\text{4}}^{\text{}}$: Almost same as $\texttt{Game}_{\text{3}}^{\text{}}$, except that ${\hat{C}}^*=g_c^{\mathsf{H_2}(x)}h_c^{\xi }$ where *x* is a randomness.

##### Lemma 3

$\texttt{Game}_{\text{0}}^{\text{}}$ and $\texttt{Game}_{\text{1}}^{\text{}}$ are indistinguishable, if $\Pi$ achieves data privacy against clouds.

##### Proof

Assume there exists a PPT adversary $\mathcal{A}$ who can distinguish $\texttt{Game}_{\text{0}}^{\text{}}$ and $\texttt{Game}_{\text{1}}^{\text{}}$ with a non-negligible advantage $\epsilon$, then we can build a PPT simulator $\mathcal{B}$ to break the data privacy against clouds of $\Pi$ with a non-negligible advantage $\epsilon '$. Note that $\Pi$ is the scheme in Yang et al. (2015).

The simulation is almost same as that in the proof of Lemma 1. To avoid repetition, we only discuss the different phases.

${{\textsc {Setup.}}}$ $\mathcal{C}$ generates a public key and returns it to $\mathcal{B}$. $\mathcal{B}$ runs ${\textsf{UKeyGen}}$ to get a cloud’s key pair $(pk_{cs}=g^{\gamma }, sk_{cs}=\gamma )$ and sends $pk_{cs}$ to $\mathcal{C}$. Then $\mathcal{B}$ generates other terms as Setup in the proof of Lemma 1.

${{\textsc {Phase 1.}}}$ Almost same as Phase 1 in the proof of Lemma 1, except that in $\mathcal{O}_{\textit{UK}}$, $\mathcal{B}$ only returns $\texttt{upk}$ to $\mathcal{A}$.

${{\textsc {Challenge.}}}$ Same as Challenge in the proof of Lemma 1.

${{\textsc {Phase 2.}}}$ $\mathcal{A}$ continues to query the oracles as Phase 1.

${{\textsc {Guess.}}}$ $\mathcal{A}$ outputs a guess $b'\in \{0,1\}$ to indicate that it plays with the game $\texttt{Game}_{b'}$. If $b'=0$, $\mathcal{B}$ outputs $b^*$ as the guess of *b*. Otherwise, $\mathcal{B}$ outputs $1-b^*$.

Similarly, if $b^*=b$, $\mathcal{B}$ has simulated $\texttt{Game}_{\text{0}}^{\text{}}$ properly; otherwise, it has simulated $\texttt{Game}_{\text{1}}^{\text{}}$. Then $\mathcal{B}$ breaks the data privacy against clouds of $\Pi$ with the advantage $\epsilon '=\epsilon$ which is non-negligible. Therefore, $\texttt{Game}_{\text{0}}^{\text{}}$ and $\texttt{Game}_{\text{1}}^{\text{}}$ are indistinguishable. $\square$

The proofs of the indistinguishability of other games are omitted, since they are same as that in the proof of data privacy against users. In $\texttt{Game}_{\text{4}}^{\text{}}$, since the information of $\texttt{m}_{b}$ is lost, the advantage of $\mathcal{A}$ is 0. Thus, ROABE achieves data privacy against clouds. Above all, we complete the proof of Theorem 1.

### Proof of Theorem 2

#### Proof

We define the following games.

- $\texttt{Game}_{\text{0}}^{\text{}}$: It is the original security game $\texttt{Game}_{\text{rvk}}^{\text{}}$.
- $\texttt{Game}_{\text{1}}^{\text{}}$: Almost same as $\texttt{Game}_{\text{0}}^{\text{}}$, except that $Y{\mathop {\leftarrow }\limits ^{\$}}{\mathbb{G}}_{\text{T}}$, $X^*={\textsc {SKE}}.{\textsf{Enc}}({\textsf{KDF}}(Y, L), \texttt{m}_b||\xi )$.
- $$\texttt{Game}_{\text{2}}^{\text{}}$$: Almost same as $$\texttt{Game}_{\text{1}}^{\text{}}$$, except that $$X^*=$$ SKE.$${\textsf{Enc}}$$$$(K, \texttt{m}_b||\xi )$$ where *K* is randomly picked from the key space of SKE.
- $\texttt{Game}_{\text{3}}^{\text{}}$: Almost same as $\texttt{Game}_{\text{2}}^{\text{}}$, except that $X^*=$ SKE.${\textsf{Enc}}$(*K*, *M*) where *M* is randomly picked from the message space of SKE and $|M|=|\texttt{m}_b|+|\xi |$.
- $\texttt{Game}_{\text{4}}^{\text{}}$: Almost same as $\texttt{Game}_{\text{3}}^{\text{}}$, except that ${\hat{C}}^*=g_c^{\mathsf{H_2}(x)}h_c^{\xi }$ where *x* is a randomness.

#### Lemma 4

$\texttt{Game}_{\text{0}}^{\text{}}$ and $\texttt{Game}_{\text{1}}^{\text{}}$ are indistinguishable, if $\Pi$ supports user revocation.

#### Proof

Assume there exists a PPT adversary $\mathcal{A}$ who can distinguish $\texttt{Game}_{\text{0}}^{\text{}}$ and $\texttt{Game}_{\text{1}}^{\text{}}$ with a non-negligible advantage $\epsilon$, then we can build a simulator $\mathcal{B}$ to break the data privacy against users of $\Pi$ with a non-negligible advantage $\epsilon '$. Note that $\Pi$ is the scheme in Yang et al. (2015). Let $\mathcal{C}$ be the challenger of $\Pi$.

Similarly, the simulation is almost same as that in the proof of Lemma 1. We only discuss the differences. In Setup, $\mathcal{C}$ only returns $pk_{cs}=g^{\gamma }$ to $\mathcal{B}$. Although $\mathcal{B}$ knows nothing about the real secret key $sk_{cs}=\gamma$ that $\mathcal{C}$ generates, $\mathcal{B}$ can randomly choose $t-1$ secret keys $\{\texttt{csk}_{i}\}_{i\in {\Phi }_{t-1}}$ and returns them to $\mathcal{A}$. Because Shamir’s secret sharing is information-theoretic secure, $\mathcal{A}$ knows nothing about $sk_{cs}$. Thus, the simulation of Setup is perfect. Other phases are almost same as that in the proof of Lemma 1, except that the restrictions of $\mathcal{T}^*$ in Challenge and ${\mathcal{R}}$ in Phase 2 are removed. Therefore, $\texttt{Game}_{\text{0}}^{\text{}}$ and $\texttt{Game}_{\text{1}}^{\text{}}$ are indistinguishable. $\square$

The proofs of the indistinguishability of other games are omitted, since they are same as that in the proof of data privacy against users. Therefore, ROABE achieves reliable user revocation. We complete the proof of Theorem 2. $\square$

### Proof of Theorem 3

#### Proof

According to the security definition, $\mathcal{A}$ wins the game if $z_{{i^*},j}=b_{{i^*},j}\cdot y_{j}+c_{{i^*},j}\ \wedge \ z^{\prime }_{{i^*},j}=b_{{i^*},j}\cdot y^{\prime }_{j}+c_{{i^*},j}.$ It is obvious that $\mathcal{A}$ can output a pair $(\texttt{ds}^*_{j,i^*},{\pi }^*_{j,i^*})$ by using $\texttt{csk}_{j}$ where $j\ne i^*$. However, since $\mathcal{A}$ does not know the verification key $vk_{i^*}=(b_{{i^*},j},c_{{i^*},j})$, the only way to find another pair $(z^{\prime }_{{j^*},i},y^{\prime }_{i})$ to satisfy the equation is randomly guessing in ${\mathbb{Z}}_{p}$ with the probability 1/*p* which is negligible. $\square$

### Proof of Theorem 4

#### Proof

Suppose there exists a PPT adversary $\mathcal{A}$ can break the verifiability with non-negligible probability, then a PPT simulator $\mathcal{B}$ can be constructed to solve DL problem. Specifically, $\mathcal{B}$ is given $(p, {\mathbb{G}}, {\mathbb{G}}_{\text{T}}, e, g, A = g^a)$ and intends to calculate $a=\log _{g}A$. The simulation is shown as follows.

${{\textsc {Setup.}}}$ $\mathcal{B}$ sets $g_c=A$ and $h_c=g^d$ where $d{\mathop {\leftarrow }\limits ^{\$}}{\mathbb{Z}}_{p}$. $\mathcal{B}$ generates other terms as the algorithm Setup and returns the public key $\texttt{pk}$ to $\mathcal{A}$.

${{\textsc {Phase 1.}}}$ Since $\mathcal{B}$ maintains the master secret key $\texttt{msk}$, it can answer all queries.

${{\textsc {Challenge.}}}$ $\mathcal{A}$ submits a message $\texttt{m}^*$ and an access tree ${\mathcal{T}}^{*}$ to $\mathcal{B}$. $\mathcal{B}$ runs Encrypt$(\texttt{pk}, \mathcal{T^*}, \texttt{m}^*)$ to return $\texttt{ct}^*$ to $\mathcal{A}$.

${{\textsc {Phase 2.}}}$ Same as Phase 1.

${{\textsc {Output.}}}$ $\mathcal{A}$ outputs a tuple {$\texttt{usk}^{* }$, ${\texttt{dct}_{1}^{*}}$, ${\texttt{dct}_{2}^{*}}$}. Specifically, $\texttt{usk}^{* }$ has been generated by $\mathcal{O}_{\textit{UK}}$ and sent to $\mathcal{A}$ in Phase 1 or Phase 2. Then $\mathcal{B}$ runs UDecrypt with $\texttt{usk}^*$ to decrypt ${\texttt{dct}_{1}^{*}}$ and ${\texttt{dct}_{2}^{*}}$ to get $({\texttt{m}_{1}^{*}},\xi _1^*)$ and $({\texttt{m}_{2}^{*}},\xi _2^*)$, respectively. $\mathcal{A}$ wins the game if ${\texttt{m}_{1}^{*}}\ne \bot \wedge {\texttt{m}_{2}^{*}}\ne \bot \wedge {\texttt{m}_{1}^{*}}\ne {\texttt{m}_{2}^{*}}$ and

$$\begin{gathered} g_{c} ^{{H_{2} (m_{1}^{*} )}} h_{c}^{{\xi _{1}^{*} }} = g^{{qH_{2} (m_{1}^{*} ) + d\xi _{1}^{*} }} \hfill \\ = \hat{C}^{*} = g_{c}^{{H_{2} (m_{2}^{*} )h_{c}^{{\xi _{2}^{*} }} }} = g^{{aH_{2} (m_{2}^{*} ) + d\xi _{2}^{*} }} \hfill \\ \end{gathered}$$

The inequality equation $\mathsf{H_2}({\texttt{m}_{1}^{*}})\ne \mathsf{H_2}({\texttt{m}_{2}^{*}})$ holds with overwhelming probability, since $\mathsf{H_2}$ is collision-resistant. Then $\mathcal{B}$ can compute

$$\begin{aligned} a=\frac{d(\xi ^*_2-\xi ^*_1)}{\mathsf{H_2}(\texttt{m}_{1}^{*})-\mathsf{H_2}(\texttt{m}_{2}^{*})} \end{aligned}$$

as the solution of the DL problem, since *d*, ${\texttt{m}_{1}^{*}}$, ${\texttt{m}_{2}^{*}}$, $\xi ^*_1$, $\xi ^*_2$ are all known to $\mathcal{B}$. $\square$
